# E4F1 and ZNF148 are transcriptional activators of the −57A > C and wild-type *TERT* promoter

**DOI:** 10.1101/gr.277724.123

**Published:** 2023-11

**Authors:** Boon Haow Chua, Nurkaiyisah Zaal Anuar, Laure Ferry, Cecilia Domrane, Anna Wittek, Vineeth T. Mukundan, Sudhakar Jha, Falk Butter, Daniel G. Tenen, Pierre-Antoine Defossez, Dennis Kappei

**Affiliations:** 1 Cancer Science Institute of Singapore, National University of Singapore, 117599 Singapore;; 2 Department of Biochemistry, Yong Loo Lin School of Medicine, National University of Singapore, 117596 Singapore;; 3 Université Paris Cité, CNRS, Epigenetics and Cell Fate, F-75013 Paris, France;; 4 NUS Center for Cancer Research, Yong Loo Lin School of Medicine, National University of Singapore, 117599 Singapore;; 5 Department of Physiological Sciences, College of Veterinary Medicine, Oklahoma State University, Stillwater, Oklahoma 74078, USA;; 6 Institute of Molecular Biology (IMB), 55128 Mainz, Germany;; 7 Institute of Molecular Virology and Cell Biology (IMVZ), Friedrich Loeffler Institute, 17493 Greifswald, Germany;; 8 Harvard Stem Cell Institute, Harvard Medical School, Boston, Massachusetts 02115, USA

## Abstract

Point mutations within the *TERT* promoter are the most recurrent somatic noncoding mutations identified across different cancer types, including glioblastoma, melanoma, hepatocellular carcinoma, and bladder cancer. They are most abundant at −146C > T and −124C > T, and rarer at −57A > C, with the latter originally described as a familial case, but subsequently shown also to occur somatically. All three mutations create de novo E26-specific (ETS) binding sites and result in activation of the *TERT* gene, allowing cancer cells to achieve replicative immortality. Here, we used a systematic proteomics screen to identify transcription factors preferentially binding to the −146C > T, −124C > T, and −57A > C mutations. Although we confirmed binding of multiple ETS factors to the mutant −146C > T and −124C > T sequences, we identified E4F1 as a −57A > C–specific binder and ZNF148 as a *TERT* wild-type (WT) promoter binder that showed reduced interaction with the −124C > T allele. Both proteins are activating transcription factors that bind specifically to the −57A > C and WT (at position 124) *TERT* promoter sequence in corresponding cell lines, and up-regulate *TERT* transcription and telomerase activity. Our work describes new regulators of *TERT* gene expression with possible roles in cancer.

The breakthrough discovery of recurrent somatic point mutations in the telomerase reverse transcriptase (*TERT*) promoter—namely, the −146C > T, −124C > T, and −57A > C mutations (positions relative to the ATG start codon) (Horn et al. 2013; Huang et al. 2013)—has highlighted the significance of noncoding mutations in the process of carcinogenesis. This exemplifies how the creation or destruction of a transcription factor binding site in regulatory elements can contribute to carcinogenesis and tumor progression through up-regulation of an oncogene or down-regulation of a tumor-suppressor gene (Rachakonda et al. 2021). The *TERT* promoter mutations (TPMs) are generally mutually exclusive and occur in >50% of bladder cancer, adult glioblastoma, hepatocellular carcinoma (HCC), and melanoma (Horn et al. 2013; Huang et al. 2013; Killela et al. 2013; Vinagre et al. 2013; Borah et al. 2015; Bell et al. 2016), roughly at par with the various *TP53* mutations as the most frequently mutated protein-coding gene (Olivier et al. 2010). Most of the point mutations take place at −146C > T and −124C > T, followed by −57A > C mutations. −57A > C was first identified as a germline mutation in a family with a high incidence of cutaneous melanoma (Horn et al. 2013) and was later found to also occur somatically (Hurst et al. 2014). Multiple other rarer variants have also been reported, for example, −124C > A and tandem CC > TT conversions at positions −138/−139 bp and −124/−125 bp (Horn et al. 2013; Huang et al. 2013; Kinde et al. 2013; Hurst et al. 2014). These mutations generally result in the creation of a de novo E26-specific (ETS) site and have been shown to up-regulate promoter activity (Horn et al. 2013; Huang et al. 2013; Bell et al. 2015). Unlike other core components of the telomerase enzyme, telomerase RNA component (*TERC*) and dyskerin pseudouridine synthase 1 (*DKC1*), that are expressed ubiquitously (Shay and Bacchetti 1997), expression of the catalytic subunit TERT is normally repressed in somatic cells. Therefore, up-regulation of *TERT* expression is associated with increased telomerase activity (Borah et al. 2015). Indeed, the majority (85%–90%) of cancers maintain their telomeres by reactivating *TERT* expression, which contributes to their indefinite proliferative potential (Hanahan and Weinberg 2011). This is in line with findings in which TPMs have been reported to be typically early events in cancers with high frequencies of these mutations (Kinde et al. 2013; Nault et al. 2013; Hunter et al. 2015; Lee et al. 2017; Lorbeer and Hockemeyer 2020), behaving as a driver mutation in cancer (Chiba et al. 2017). *TERT* activation can also occur as a result of constitutive oncogenic cell signaling pathways, including TGFB/SMAD signaling and WNT/CNTB1 signaling. Both can lead to up-regulation of MYC, which binds E-boxes found in the *TERT* promoter to drive *TERT* expression (Wu et al. 1999; Casillas et al. 2003; Ge et al. 2010). Likewise, SP1 can bind to five SP1 sites found in the *TERT* promoter and synergizes with MYC to increase *TERT* transcription (Liu et al. 2007; Kyo et al. 2008). These processes themselves are tightly regulated as illustrated by the KAT5-dependent acetylation of SP1, which inhibits SP1 binding to the *TERT* promoter and results in *TERT* repression (Rajagopalan et al. 2017). Furthermore, unlike conventional promoters, the majority of the CpG islands in the *TERT* promoter region are hypermethylated in cancer (Dessain et al. 2000; Renaud et al. 2007; Stern et al. 2017) except for a small nonmethylated region upstream of the transcriptional start site (TSS), which coincides with the TPMs. Hypermethylation prevents the binding of transcriptional repressors such as CTCF and E2F1, whereas the nonmethylated region grants access to activating transcription factors (Renaud et al. 2007; Zhang et al. 2014). In comparison, noncancerous primary cell lines are generally hypomethylated across the CpG islands (Renaud et al. 2007; Esopi et al. 2020). *TERT* is also often expressed in a monoallelic manner, both in wild-type (WT) and TPM cell lines, with hypermethylation of promoter sequences being associated with repression and unmethylated alleles being expressed (Esopi et al. 2020). In consequence, the expressed mutant allele in heterozygous TPM cell lines is associated with active histone marks such as H3K4me2/3, whereas the transcriptionally silenced WT allele is associated with repressive histone H3K27me3 marks (Stern et al. 2015, 2017).

As mentioned, TPMs result in the creation of a de novo site for ETS, a transcription factor family that consists of 29 genes (Wang and Zhang 2009) that generally recognize the same GGA(A/T) motif (Hollenhorst et al. 2007). Bell et al. (2015) systematically tested 13 of the ETS family members that are expressed in glioblastoma multiforme (GBM) to determine which ETS member(s) was/were responsible for the up-regulation of *TERT* expression in −146C > T and −124C > T-containing GBM cell lines. Here, GA binding protein transcription factor subunit alpha (GABPA) was shown to be the only critical member in the up-regulation of the *TERT* gene specific to mutant −146C > T and −124C > T cell lines. GABP (GABPA + GABPB) is the only obligate multimeric factor among the ETS family members (Oikawa and Yamada 2003) and is proposed to bind one of the two slightly overlapping endogenous ETS sites (ETS-96 and ETS-91) (Fig. 1A) alongside the de novo ETS site created by the −146C > T and −124C > T mutations. The same group would later show that knockdown of *GABPB1L*, the long isoform of *GABPB1* (involved in GABP dimerization), also affected *TERT* expression in the −146C > T and −124C > T GBM cell lines but not in WT cell lines (Mancini et al. 2018). Other factors that have been reported to regulate *TERT* expression in a TPM-dependent manner include NFKB2 and ETS1/2 (−146C > T mutants only) (Li et al. 2015), as well as phosphorylated ETS1 (owing to constitutive MAPK pathway caused by NRAS/BRAF mutations in melanoma) (Vallarelli et al. 2016).

**Figure 1.**
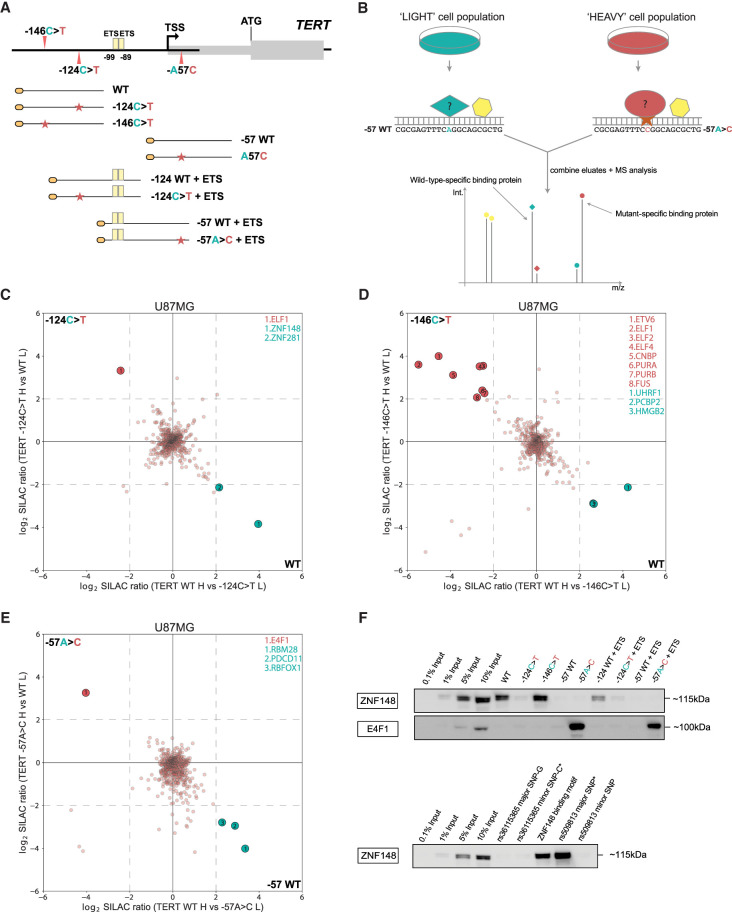
ZNF148 and E4F1 bind to WT and −57A > C *TERT* promoter sequence in vitro. (*A*) Schematic showing the coverage of the *TERT* promoter sequence by the DNA probes used in this study. (*B*) Workflow for a quantitative SILAC-based protein–DNA interaction screen. Biotinylated concatenated DNA oligonucleotides from *A* were immobilized on magnetic streptavidin beads, and bound proteins were detected by MS or western blot. −57WT and −57A > C probes are shown as an example of WT and mutant sequences. Specific protein binders display a differential SILAC ratio, with background binders showing a 1:1 ratio. (*C*) Two-dimensional interaction plot for WT versus −124C > T using SILAC-labeled U87MG nuclear protein extracts. Mutant-specific binders are labeled in red, and WT-specific binders are labeled in green. (*D*) Two-dimensional interaction plot for WT versus −146C > T. Mutant- and WT-specific binders are labeled similar to those in *C*. (*E*) Two-dimensional interaction plot for WT versus −57A > C. Mutant- and WT-specific binders are labeled similar to those in *C*. (*F*) Sequence specific pull-down of endogenous ZNF148 and E4F1 with HeLa nuclear extracts using the nine probes shown in *A*, *top*. Sequence-specific pull-down of endogenous ZNF148 with HeLa nuclear extracts with probes for rs36115365 major SNP-G and minor SNP-C, rs509813 major and minor SNP, and *CDKN1A* promoter (containing ZNF148 binding site) probes (*bottom*). Please note the occurrence of a double band for endogenous ZNF148, which may be owing to isoforms and/or post-translational modifications.

Although the aforementioned studies have managed to elucidate partly how TPMs can alter *TERT* transcription, we here aimed to identify additional differential binders to WT and mutant *TERT* promoters by systematic proteomics screening in order to provide additional mechanistic insights underlying *TERT* expression control.

## Results

### ZNF148 and E4F1 bind to the WT and −57A > C *TERT* promoter, respectively, in vitro

We reasoned that a systematic screen would identify additional differential binders to either the WT or mutant (−146C > T, −124C > T, and −57A > C) *TERT* promoter. We first designed different DNA probes, each containing either the WT or mutant *TERT* promoter sequence (Fig. 1A; for exact sequences, see Supplemental Table S1). We then performed in vitro DNA reconstitution pull-downs with the WT and mutant probes combined with SILAC-based quantitative mass spectrometry analysis (Fig. 1B) with either light-labeled or heavy-labeled U-87MG (containing the −124C > T mutation) (Supplemental Table S2) nuclear protein extracts. Corresponding eluates were combined and analyzed using quantitative mass spectrometry to identify proteins that were binding preferentially to either the WT (bottom right quadrant) or mutant (top left quadrant) *TERT* promoter sequences (Fig. 1C–E). By performing the DNA pull-downs in both a “forward” (heavy-labeled SILAC extracts applied to the mutant sequence and light-labeled SILAC extracts applied to the WT sequence) and a “reverse” experiment (the SILAC-labeled extracts were applied to the opposite DNA probes), reciprocal SILAC ratios confirm genuine enrichment and exclude common contaminants from unlabeled proteins such as keratins (bottom left) or labeling artifacts (top right). Only proteins specifically enriched with SILAC ratios greater than four in both the “forward” and “reverse” experiments were considered to be positive hits (Fig. 1C–E; Supplemental Tables S3–S5) as a common cut-off (Butter et al. 2010, 2012; Kappei et al. 2013; Makowski et al. 2016; Fang et al. 2017) to ensure a robust relative binding preference.

Consistent with previous reports (Horn et al. 2013; Huang et al. 2013) that predicted the creation of a de novo ETS motif, several ETS factors were identified binding preferentially to −124C > T (ELF1) and −146C > T (ELF1, ELF2, ELF4, ETV6) mutant sequences (Fig. 1C,D; Supplemental Tables S3, S4). GABPA was not identified, consistent with a similar screen that required the introduction of a second ETS site to observe GABPA binding (not included in our probes used for mass spectrometry analysis) (Makowski et al. 2016). Although the −57A > C mutation also creates an ETS binding motif, no ETS factors were identified binding preferentially to the mutant sequence. Instead, we identified E4F1 as the only −57A > C-specific candidate protein (Fig. 1E; Supplemental Table S5). The design of our experiment allowed us to read out both mutant- and WT-specific binding proteins simultaneously. In agreement with a recent report (Mondal et al. 2022), ZNF148 and ZNF281 were specifically enriched on the WT probe in comparison to the −124C > T mutant, as well as UHRF1 on the WT probe compared with −146C > T (Fig. 1C,D; Supplemental Tables S3, S4). Notably, some known single-stranded DNA-/RNA-binding proteins (including CNBP, PURA, PURB, FUS, PCBP2, HMGB2, RBM28, PDCD11, and RBFOX1) were also identified to preferentially bind to either the WT or mutant sequence (Fig. 1D,E; Supplemental Tables S4, S5), which may be owing to the presence of some single-stranded DNA as part of our concatenated DNA probe preparation. We next focused on proteins with a DNA binding domain that are more likely to bind directly to the dsDNA sequences.

To systematically validate the absolute enrichment of all WT or mutant specific binders, HeLa (WT *TERT* promoter) (Supplemental Table S2) nuclear protein extracts were incubated with the five probes used above, alongside −124WT + ETS/−124C > T + ETS and −57WT + ETS/−57A > C + ETS (Fig. 1A), followed by western blot. The additional probes contain the two endogenous ETS sites between −99 and −89. Although a previous report (Makowski et al. 2016) using a similar pull-down approach had reported GABPA binding in the presence of both the endogenous ETS sites and de novo ETS sites created by the −124C > T mutation using UACC903 (melanoma; −124C > T mutant) nuclear extracts (Fig. 1A; Supplemental Table S1), we could not detect GABPA enrichment to any of the nine probes using antibodies against endogenous GABPA (Supplemental Fig. S1A), consistent with our mass spectrometry results. Similar results were obtained for pull-downs with A375 (melanoma, −146C > T mutant) (Supplemental Table S2) nuclear extracts or with HeLa cells transiently transfected with both N-terminally and C-terminally tagged GFP-GABPA. In all cases, we were unable to enrich for GABPA (Supplemental Fig. 1A), which might be partly attributed to cell type–specific effects. In contrast, N-terminally tagged GFP-ELF1 showed strong absolute enrichment on the −146C > T probe, whereas N-terminally tagged GFP-ELF2 showed strong absolute enrichment on all mutant probes compared with their respective WT (Supplemental Fig. S1A), confirming the idea that ETS family members may represent the predominant transducers of −124C > T-dependent and −146C > T-dependent *TERT* expression. The fact that they were enriched in both our mass spectrometry and western blot experiments in the absence of the additional endogenous ETS sites suggests that their binding, at least in vitro, only requires the TPM alleles.

Furthermore, endogenous ZNF148 was enriched on the WT sequence (WT and −124WT + ETS) but showed reduced interactions with the −124C > T and −124C > T + ETS mutant probes (Fig. 1F). ZNF148 has been reported to be an allele-specific transcription factor in two previous studies (Butter et al. 2012; Fang et al. 2017), shown to preferentially bind one single-nucleotide polymorphism (SNP) allele over the other. We decided to include both SNPs in our probes design (rs509813 and rs36115365) alongside a known ZNF148 binding site (*CDKN1A* promoter) (Supplemental Table S4; Fang et al. 2017). Differential ZNF148 binding to the rs36115365 SNP was particularly intriguing because it is located 18 kb upstream of the 5′-end of *TERT*, and *ZNF148* mRNA knockdown in a panel of pancreatic cancer cell lines resulted in reduced *TERT* expression. However, we could not recapitulate binding to the rs36115365 SNP using HeLa nuclear extracts, although we could readily see differential binding of ZNF148 to the rs509813 major SNP (unrelated to the *TERT* gene, located on Chromosome 11 in the promoter of *CHRM1*) and the *CDKN1A* promoter binding site in the same pulldown assay (Fig. 1F). Although the lack of ZNF148 binding to the rs36115365 may again in part be attributed to cell type–specific differences, the identification of ZNF148 binding to the proximal promoter sequence might implicate it in direct *TERT* mRNA expression regulation independent of long-range chromatin–chromatin interactions. Although ZNF281 displayed enrichment to some of the probes (Supplemental Fig. S1A), the enrichment was inconsistent between endogenous and GFP-tagged ZNF281 and did not readily recapitulate the original mass spectrometry data. Therefore, ZNF281 was excluded from functional downstream analyses.

Finally, we could validate E4F1 binding to the −57A > C mutant sequence using an antibody against endogenous E4F1 on both the −57A > C and −57A > C + ETS probes compared with their respective WT controls with strong absolute enrichment (Fig. 1F). These data collectively show that ZNF148 and E4F1 specifically bind to the WT and −57A > C *TERT* promoter sequences in vitro, respectively.

### ZNF148 and E4F1 bind directly to WT and −57A > C *TERT* promoter, respectively

**Figure 2.**
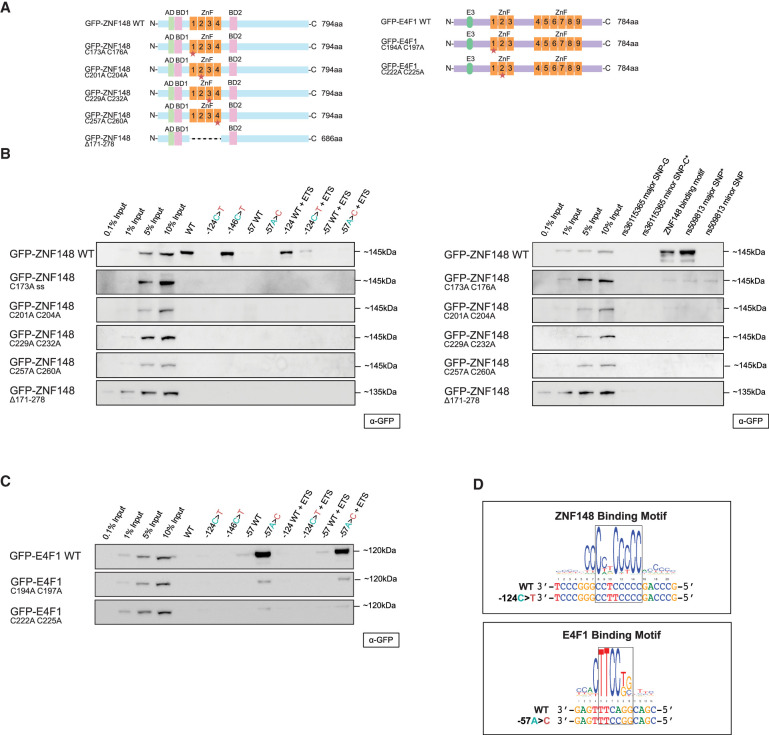
ZNF148 and E4F1 require their DNA binding domains to bind in vitro to WT and −57A > C *TERT* promoter, respectively. (*A*) Schematic representation of ZNF148 and E4F1 WT and DNA binding mutant constructs. (*B*) Sequence-specific pull-down of recombinant GFP-tagged WT or binding mutant of ZNF148 with HeLa nuclear extracts with the nine probes shown in Figure 1A (*left*) alongside rs36115365 major SNP-G and minor SNP-C, rs509813 major SNP and minor SNP, and *CDKN1A* promoter (containing ZNF148 binding site) (*right*). (*C*) Sequence-specific pull-down of recombinant GFP-tagged WT or binding mutant of E4F1 with HeLa nuclear extracts with the nine probes shown in Figure 1A. (*D*) Binding motif of ZNF148 and E4F1 aligned to the *TERT* promoter sequence based on the consensus motif derived from the MethMotif database (Xuan Lin et al. 2018).

To test whether ZNF148 and E4F1 bind directly to the respective *TERT* promoter sequences, DNA binding mutant constructs of both ZNF148 and E4F1 were generated by mutating key cysteine residues in the C2H2 zinc finger motifs in both proteins and were then tested in our DNA pull-down assay (Fig. 2A; Le Cam et al. 2006). Although GFP-ZNF148 WT displayed the same binding pattern as seen for the endogenous protein, mutation in any of the four zinc fingers or deletion of all four zinc fingers in ZNF148 resulted in the loss of ZNF148 binding to the WT, −146C > T, −124WT + ETS, and ZNF148 binding motif probes (Fig. 2B). Similarly, although GFP-E4F1 WT was again enriched on both the −57A > C and −57A > C + ETS probes, mutation in either of the first two zinc fingers in E4F1, previously shown to be critical for its DNA binding ability (Rooney et al. 1998), resulted in the loss of E4F1 binding (Fig. 2C). These results suggest that both factors likely bind the *TERT* promoter directly. We also aligned the respective *TERT* promoter alleles to the binding motifs of ZNF148 and E4F1 as predicted by the MethMotif database (Xuan Lin et al. 2018) and identified a high degree of overlap between the general motifs and the exact binding sites in the *TERT* promoter. Importantly, the mutations overlap with key residues, in which alteration of one base results in the disruption (−124C > T) and creation (−57A > C) of the ZNF148 and E4F1 binding motif, respectively (Fig. 2D). These data further strengthen a model in which ZNF148 and E4F1 bind directly to the WT and −57A > C mutant *TERT* promoter sequences.

### ZNF148 and E4F1 do not affect *TERT* promoter methylation

Transcription factors can alter gene expression through a myriad of mechanisms, for example, by changing chromatin access, recruiting epigenetic modifiers both at the DNA or histone level, and impacting polymerase recruitment and regulation (Lambert et al. 2018). Given the nonconventional DNA methylation pattern at the *TERT* locus with hypermethylation around the TPM sites in cancer cells and hypomethylation in noncancerous primary cells (Dessain et al. 2000; Renaud et al. 2007; Stern et al. 2017), we next tested whether the DNA methylation pattern is altered between cell lines with different TPM status. To this end, we conducted pyrosequencing using gDNA extracted from the different cell lines. In agreement with the above, the DNA methylation pattern at the CpG islands across the *TERT* promoter is differently dependent on the mutation status of the cell lines. In general, WT cell lines (HeLa Kyoto and 253J, excluding HCT116, which displays hypomethylation) display a higher degree of methylation compared with cell lines with either −124C > T (T24 and U87MG) or −146C > T (A375) mutation (Supplemental Fig. S2). The two cell lines with the −57A > C mutation displayed more variation in their DNA methylation levels, with JON showing low DNA methylation pattern across its *TERT* promoter, which is closer to IMR-90 (immortalized normal fibroblasts, in which normal cells typically display hypomethylation across the *TERT* promoter), whereas 575A is closer to WT cell lines. Of note, we observed a drop in DNA methylation levels especially around the region where both −124C > T and −146C > T mutation reside (between CG7 and CG10, which includes the third SP1 site and near the second and fourth SP1 sites) in all cell lines despite their mutation statuses. In comparison, the decrease in DNA methylation levels is not as pronounced for the −57A > C mutation locus (between CG17 and CG18) across all cell lines. These data may indicate generally greater accessibility at the key binding sites for TPM-specific transcription factors (Horn et al. 2013; Huang et al. 2013; Stern et al. 2015, 2017).

**Figure 3.**
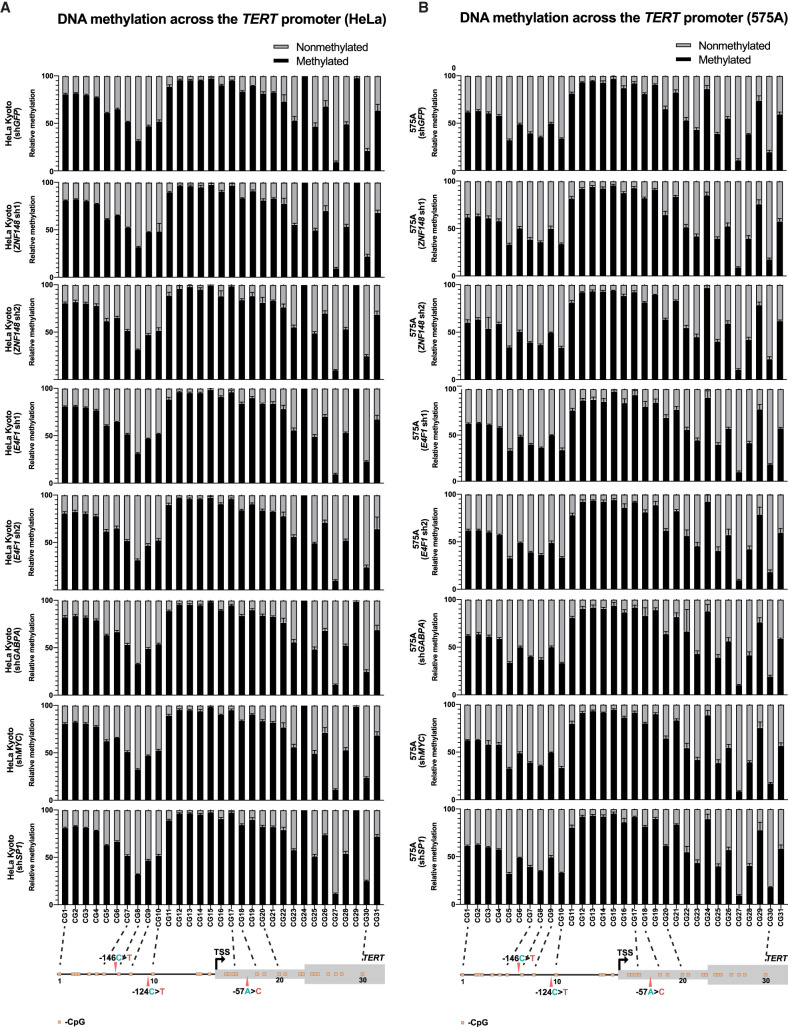
*TERT* promoter mutant–specific binders do not affect the promoter methylation status. (*A*) Relative DNA methylation frequency of CpGs across the *TERT* promoter in HeLa Kyoto cells following shRNA knockdown. (*B*) Relative DNA methylation frequency of CpGs across the *TERT* promoter in 575A cells following shRNA knockdown.

To test if ZNF148 and E4F1 might affect the epigenetics of the *TERT* promoter, pyrosequencing was performed to study the effects on DNA methylation following shRNA knockdown. No significant differences were observed in DNA methylation of the proximal *TERT* promoter following shRNA knockdown of *ZNF148* mRNA in HeLa Kyoto and of *E4F1* mRNA in 575A (Fig. 3A,B). In addition, no effects on DNA methylation were observed following the knockdown of other known transcription factors of the *TERT* promoter (*MYC*, *SP1*, *GABPA*, *GABPB1L*) in the two cell lines (Fig. 3A,B), suggesting that these factors do not act on the *TERT* promoter by modulating the DNA methylation status.

### ZNF148 and E4F1 act as transcriptional activators of *TERT*

**Figure 4.**
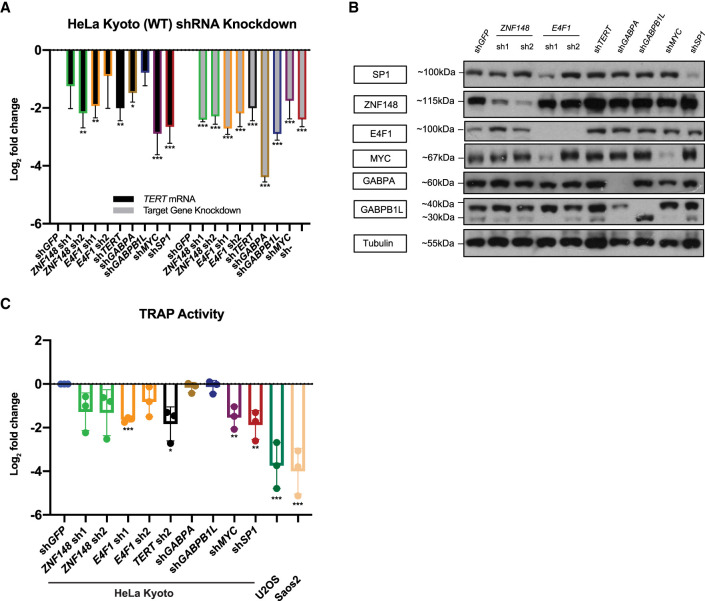
ZNF148 knockdown leads to reduction of *TERT* promoter transcription and telomerase activity in HeLa cells. (*A*) mRNA expression data of *TERT* and target genes following 48 + 72 h (48-h virus transduction, 72-h puromycin selection) post-shRNA knockdown in HeLa, with sh*GFP* as control. Data shown as the mean of values from three biological replicates. (*B*) Representative western blot image following 48 + 72 h post-shRNA knockdown in HeLa, with sh*GFP* as control. (*C*) TRAP assay measuring telomerase activity following 48 + 72 h post-shRNA knockdown in HeLa, with sh*GFP* as control. Telomerase-negative U2OS and Saos2 were used as negative controls. Data shown as mean of values from three biological replicates. All statistical significance was calculated using a two-sampled *t*-test, and the degree of significance is indicated as follows: (*) *P* < 0.05, (**) *P* < 0.01, (***) *P* < 0.001.

**Figure 5.**
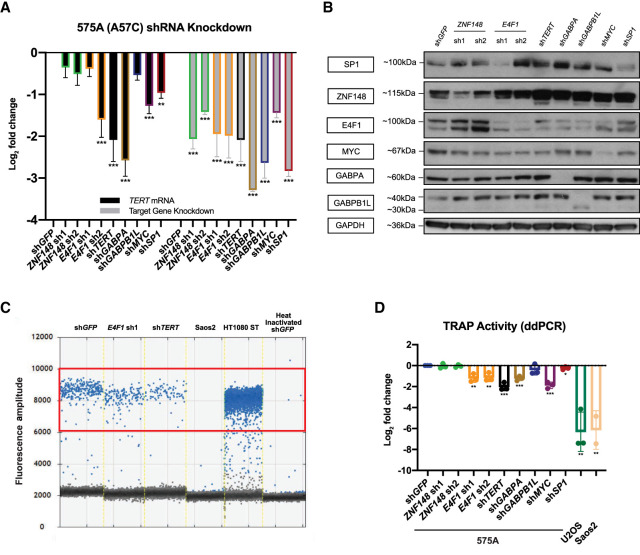
*E4F1* knockdown leads to reduction of *TERT* promoter transcription and telomerase activity in 575A cells. (*A*) mRNA expression data of *TERT* and target genes following 48 + 72 h (48-h virus transduction, 72-h puromycin selection) post-shRNA knockdown in 575A, with sh*GFP* as control. Data shown as mean of values from three biological replicates. (*B*) Representative western blot image following 48 + 72 h post-shRNA knockdown in 575A, with sh*GFP* as control. (*C*) ddPCR-TRAP assay measuring telomerase activity following 48 + 72 h post-shRNA knockdown in 575A, with sh*GFP* as control. Telomerase-negative U2OS and Saos2 were used as negative controls. Positive droplets quantified are labeled in blue. (*D*) Quantification of data in *C*; data shown as mean of values from three biological replicates. All statistical significance was calculated using a two-sampled *t*-test, and the degree of significance is indicated as follows: (*) *P* < 0.05, (**) *P* < 0.01, (***) *P* < 0.001.

To test if the promoter binding of ZNF148 and E4F1 translates into an actual effect on *TERT* transcript levels, we depleted both factors with two shRNAs each (Fig. 4, 5; Supplemental Fig. S3) across a panel of cell lines with either WT or mutant *TERT* promoters (Supplemental Table S2). In addition, we used shRNAs targeting *TERT*, *GABPA*, *GABPB1L*, *MYC*, and *SP1* mRNAs as positive controls. MYC and SP1 are factors that have previously been reported to bind to the E-box and SP1 sites on the *TERT* promoter (Cong et al. 1999; Horikawa et al. 1999; Takakura et al. 1999) and affect *TERT* transcription, whereas knockdown of *GABPA* and *GABPB1L* have been reported to only affect *TERT* transcription in cell lines containing the −124C > T or −146C > T mutation (Bell et al. 2015; Mancini et al. 2018). Upon *ZN148* mRNA knockdown in HeLa cells, we observed a reduction of *TERT* expression for both shRNAs (Fig. 4A). As expected, sh*TERT*, sh*MYC*, and sh*SP1* also resulted in reduction of *TERT* expression. Albeit HeLa cells were *TERT* promoter WT, knockdown of *GABPA* mRNA also led to a statistically significant reduction of *TERT*. It is of note that knockdown of all other factors excluding ZNF148 resulted in an increase in *ZNF148* mRNA and ZNF148 protein levels (Fig. 4B; Supplemental Fig. S3A), suggesting some degree of interdependency or feedback regulation. Because we could not identify a commercially available TERT antibody that showed depletion of a specific band upon sh*TERT* transduction, we used the telomerase repeat amplification protocol (TRAP) assay as an indirect measure for changes in TERT protein levels. HT1080 super telomerase cells (HT1080 STs) were used as a positive control, whereas the telomerase-negative cell lines U2OS and Saos2 were used as negative controls alongside heat-inactivated cell extracts. Using HeLa Kyoto cells, we observed a reduction of telomerase activity for both shRNAs targeting *ZNF148*, whereas there was a marginal effect caused by sh*GABPA* and sh*GABPB1L*, consistent with previous reports in which these factors only affected cell lines with −146C > T or −124C > T mutations. Similarly, we also saw a reduction in telomerase activity with our positive controls sh*TERT*, sh*MYC*, and sh*SP1* (Fig. 4C; Cong and Bacchetti 2000; Takakura et al. 2001; Hou et al. 2002; Choi et al. 2010). We also observed a similar trend of reduction in *TERT* mRNA and telomerase activity in another WT *TERT* promoter cell line, 253J (Supplemental Table S1), following *ZNF148* shRNA knockdown (Supplemental Fig. S3B,C), concomitant with a reduction in *TERT* expression and telomerase activity upon *TERT*, *MYC*, and *SP1* knockdown. Again, both *GABPA* and *GABPB1L* knockdown had marginal effects on *TERT* mRNA expression, although sh*GABPB1L* led to a more pronounced reduction in telomerase activity. In contrast, *ZNF148* knockdown in −124C > T-positive T24 cells (Supplemental Table S1) had marginal effects on *TERT* mRNA expression and telomerase activity, in agreement with a lack of promoter binding in the presence of −124C > T mutations (Supplemental Fig. S3D,E), whereas our positive controls sh*TERT* and sh*MYC* both resulted in a consistent reduction of *TERT* mRNA expression and telomerase activity.

In −57A > C-positive 575A cells (Supplemental Table S7), in addition to sh*TERT* and sh*MYC*, knockdown of E4F1 resulted in a general reduction of *TERT* mRNA expression (Fig. 5A,B) and TRAP activity as measured by digital droplet PCR (ddPCR) (Ludlow et al. 2014, 2018). The latter showed a clear reduction of positive droplets for sh*E4F1* and sh*TERT* compared with sh*GFP* (control), with an abundance of droplets in HT1080 STs and absence of droplets for U2OS and Saos2 alongside heat-inactivated sh*GFP* (Fig. 5C,D). Therefore, the knockdown of *ZNF148* in a WT *TERT* promoter cell line (HeLa Kyoto) and the knockdown of *E4F1* in the presence of the −57A > C mutation resulted in reduction of telomerase activity. These data show a functional effect of these transcription factors at their respective *TERT* promoter loci and are in line with allele-specific regulation.

## Discussion

We showed that a SILAC-based DNA–protein interaction assay could be a highly viable and robust method for the identification of novel binding factors to WT and mutant sequences. The binding of ETS factors such as ELF1 and ELF2 to the de novo ETS site created by the mutant sequences (Supplemental Fig. S1A) here serves as a proof of concept. Although all three TPMs create the same basic ETS consensus site, no ETS factors were enriched on the mutant −57A > C *TERT* promoter (Fig. 1E) in our proteomics screen. This may be because of the differences in flanking sequences between the −57A > C mutant (gccGGAAactc) and the −124C > T and −146C > T mutants (cccGGAAgggg) despite the common GGAA motif, in agreement with previous reports showing the importance of flanking sequences in determining which ETS family members are recruited to specific loci (Wei et al. 2010).

Next, we were unable to reproduce the enrichment of GABPA using probes that contain the endogenous ETS sites alongside the de novo −124C > T mutation as previously reported (Makowski et al. 2016). We were also unable to recapitulate the enrichment of ZNF148 on the minor rs36115365 SNP using the exact same pulldown assay as a previous report (Fang et al. 2017), which might be partly attributed to different wash conditions (e.g., buffer salt concentration) and incubation duration, as well as cell line–specific effects. In the latter study, the investigators showed that knockdown of *ZNF148* led to a reduction of *TERT* transcription in both pancreatic cancer cell lines containing the minor SNP and those with the major SNP. This lends support to our work, implying ZNF148 as a direct transcriptional activator at the proximal WT *TERT* promoter. If ZNF148 binding to the rs36115365 is context specific, binding to both the distal variant and the proximal promoter could lead to a ZNF148-mediated chromatin–chromatin interaction to further strengthen the transcriptional output. Similar considerations might apply to the lack of ETS factors identified for the −57A > C mutation. In addition, given that all TPMs create a basic ETS motif, the exact nucleotide context of the −57A > C mutation might further explain these differences.

In contrast, in this study, we have identified an −57A > C TPM-specific transcriptional activator: E4F1 has been previously reported to be involved in carcinogenesis by regulating viral oncoprotein expression (Lee and Green 1987; Fernandes and Rooney 1997) and TP53 ubiquitination (Le Cam et al. 2006). It also directly controls the transcription of multiple mitochondrial and checkpoint protein genes, including DNA-damage response protein CHEK1 (Rodier et al. 2015). The oncogenic behavior is consistent with our study, in which the −57A > C TPM creates a de novo binding site for E4F1 to up-regulate *TERT* oncogene expression. Furthermore, based on our knockdown experiments, we postulate that there might be possible cross talk between the different transcription factors that regulate *TERT* expression. For instance, the up-regulation of ZNF148 following knockdown of all other factors, including *E4F1*, could be a compensatory mechanism to up-regulate *TERT* expression (Figs. 4B, 5B), which is vital for unlimited cancer cell proliferation.

In conclusion, we have identified two novel transcriptional regulators of the *TERT* locus that contribute to *TERT* up-regulation in a mutation-dependent manner. Specifically, we could identify ZNF148 as an additional factor that could regulate the *TERT* promoter at the −124 position, with a corresponding decrease in *TERT* expression and telomerase activity upon *ZNF148* knockdown in WT *TERT* promoter cell lines. Additionally, we here report the first transcription factor that specifically regulates *TERT* expression at the −57A > C mutant promoter. Our study alongside others (Butter et al. 2010; Makowski et al. 2016; Fang et al. 2017; Liu et al. 2017) shows the potential of quantitative mass spectrometry in combination with in vitro reconstitution as a systematic strategy to interpret noncoding mutations.

## Methods

### Cell culture and SILAC labeling

All cell lines (A375, HCT116, HeLa Kyoto, Saos2, U2OS, U87MG, 253J, T24, 575A, JON, HT1080 ST [Cristofari and Lingner 2006], IMR-90, and HEK293T) used in this study were cultured in 4.5 g/L glucose Dulbecco's Modified Eagle Medium (DMEM), supplemented with 10% FBS and 100 U/mL penicillin, 100 μg/mL streptomycin (Gibco). Cell lines were maintained at 37°C with 5% CO_2_ in a humidified incubator. All cell lines were authenticated by STR profiling (first BASE) and regularly tested negative for mycoplasma using the MycoAlert plus detection kit (Lonza).

For SILAC labeling, U87MG cells were cultured in DMEM (-Arg, -Lys) for SILAC (Thermo Fisher Scientific), supplemented with 10% dialyzed FBS (PAN-Biotech) and 100 U/mL penicillin, 100 μg/mL streptomycin (Gibco), in addition to either nonlabeled 42 mg/L ^12^C_6_^14^N_4_-arginine and 73 mg/L ^12^C_6_^14^N_2_-lysine or heavy-labeled 42 mg/L ^13^C_6_^15^N_4_-arginine and 73 mg/L ^13^C_6_^15^N_2_-lysine. U87MG cells were cultured for at least 2 wk in SILAC media before experiments to ensure an incorporation rate of >98%.

Ideal antibiotic concentrations for plasmid selection were optimized for each cell line by generating killing curves. The eventual concentrations used were 0.5 μg/mL for U87MG, 1 μg/mL for HeLa Kyoto, 575A, 2 μg/mL for T24, and 2.5 μg/mL puromycin for 253 J; 10 μg/mL blasticidin S was used for 575A, and 300 μg/mL hygromycin B were used for 575A.

### Sanger sequencing of cell lines

Genomic DNA (gDNA) was extracted from various cell line pellets using the QIAamp DNA blood mini kit (Qiagen) according to the manufacturer's instructions. The *TERT* promoter was then amplified with PCR using corresponding primers with the Q5 DNA polymerase (NEB) (Supplemental Table S6). PCR conditions were as follows: 30 sec at 98°C; 35 cycles of 10 sec at 98°C, 25 sec at 61°C, and 40 sec at 72°C. The PCR products were purified using the QIAquick PCR purification kit (Qiagen) according to the manufacturer's protocol before being submitted for Sanger sequencing (AITBiotech). Chromatograms were inspected using 4Peaks (Nucleobytes).

### Plasmids and cloning

Full-length ZNF148, E4F1, ELF1, ELF2, GABPA, and ZNF281 were amplified from HeLa Kyoto or U2OS cDNA (RNeasy plus mini kit; Qiagen) using the Q5 DNA polymerase (NEB) with specific primers (Supplemental Table S6). These sequences were cloned into the TOPO TA entry vector (Thermo Fisher Scientific) before LR recombination into gateway-compatible expression vectors: pcDNA3.1 with N-terminal GFP tag or pcDNA-DEST47 with C-terminal GFP tag. Point mutants were generated using the Q5 site-directed mutagenesis kit (NEB) coupled with specific primers (Supplemental Table S6). Sequences for shRNA oligonucleotides were shortlisted from the GPP web portal (BROAD Institute; https://portals.broadinstitute.org/gpp/public/gene/search) based on high specificity criteria. Primers (Supplemental Table S7) were annealed, phosphorylated, and cloned into pLKO.1 vector following restriction enzyme digest and DNA ligation.

### Transfection and transduction

Four million to 4.5 million HeLa Kyoto cells were seeded per 15-cm dish the evening before transfection for plasmid transfections. The cells were transfected with 121.6 μL of 1 mg/mL of linear polyethylenimine (PEI; MW 25,000; Polysciences) and 30.4 μg of plasmid DNA diluted in Opti-MEM (Gibco). Media was replaced with fresh culture medium after 7 h, and cells were harvested 48 h post transfection.

For lentiviral production, 300,000 HEK293T were seeded per well in a six-well plate format the evening before transfection. Cells were transfected with 2.5 μL of 1 mg/mL PEI, 0.5 μg of transfer vector (viral genome and gene of interest), 0.25 μg each of pMD2.G envelope plasmid (VSV-G), pRSV-Rev packaging plasmid (Rev), and pMDLg/pRRE packaging plasmid (Gag and Pol) diluted in Opti-MEM. Media was changed after 24 h, and cell supernatants were harvested using a syringe and 0.45-μm filter unit 72 h post transfection.

For viral transduction, 100,000–200,000 cells were seeded per well in six-well plates the evening before transduction. For every 1 mL of virus supernatant, 10 μL of 1 M HEPES (final 10 mM; Sigma-Aldrich) and 1 μL of 10 mg/mL (final 10 μg/mL) polybrene were added before addition to the cells to be transduced. These were replaced with fresh media 24 h post transduction, and selective media (puromycin, hygromycin, or blasticidin) were added 48 h post transduction for cell selection.

### Reverse transcription and qPCR

Cells were harvested after 48 h (lentiviral transduction) + 72 h (puromycin selection). Cell pellets were washed with 1× PBS and RNA were extracted using the RNeasy plus mini kit (Qiagen) according to the manufacturer's protocol. Total RNA was eluted in 30 μL nuclease-free water and used for cDNA conversion with the SuperScript IV reverse transcriptase (Thermo Fisher Scientific) based on the manufacturer's protocol.

Each qPCR reaction consisted of 5 μL of 2× QuantiNOVA SYBR Green PCR master mix (Qiagen), 0.5 μL of 10 μM forward and reverse primers each, alongside the appropriate cDNA amounts, topped up to 10 μL with nuclease-free water. Each reaction was set up in triplicates and ran on a QuantStudio3 or QuantStudio5 machine (Applied Biosystems), with the following protocol used: 2 min at 50°C, 10 min at 95°C, followed by 40 cycles at 95°C for 15 sec and 60°C for 1 min, and then, finally, 15 sec at 95°C and 1 min at 60°C. mRNA levels of *TBP* were used as a housekeeping reference for the normalization of mRNA levels of target genes (Supplemental Table S8).

### Nuclear protein extraction

Cells were harvested and washed in PBS and incubated on ice for 10 min with five times the pellet volume of cold buffer A (10 mM HEPES at pH 7.9, 10 mM KCl, 1.5 mM MgCl_2_). Cells were pelleted again and resuspended in approximately two times the pellet volume of cold buffer A + (buffer A supplemented with 0.1% IGEPAL CA-630, 0.5 mM DTT, and 1× cOmplete protease inhibitor; Merck) and homogenized in a glass Dounce homogenizer (type B pestle) to mechanically lyse the cell cytoplasm. HeLa Kyoto cells required 40 Dounces, whereas 575A and U-87MG needed 30 Dounces for maximum cytoplasmic lysis while keeping most nuclei intact. The supernatant (cytoplasmic fraction) was disposed as it was not required for this study. The nuclei were washed once with PBS and were then resuspended in 1.7× pellet volume of buffer C + (20% v/v glycerol, 420 mM NaCl, 20 mM HEPES at pH 7.9, 2 mM MgCl_2_, 0.2 mM EDTA at pH 8, 0.1% IGEPAL CA-630, 0.5 mM DTT, and 1× cOmplete protease inhibitor; Merck) before being incubated on a rotating wheel at 30–35 rpm for 1 h at 4°C (cold room) in order to extract nuclear proteins. The suspension was then centrifuged at maximum speed for 1 h at 4°C to collect the nuclear extract. Protein quantification was performed with the Pierce BCA protein assay kit (Thermo Fisher Scientific) according to the manufacturer's protocol, and the nuclear protein extracts were diluted to the required concentrations using buffer C+.

### In vitro reconstitution DNA pull-down

DNA in vitro reconstitution pull-downs were essentially performed as previously described (Kappei et al. 2017). Twenty-five microliters of 100 μM respective forward- and reverse-strand oligonucleotides (Supplemental Table S1) were denatured at 80°C and reannealed via gradual cooling. Double-stranded oligonucleotides were then concatemerized with 100 U T4 polynucleotide kinase (Thermo Fisher Scientific) and 20 Weiss U T4 DNA ligase (Thermo Fisher Scientific), biotinylated with desthiobiotin-ATP (Jena Bioscience) by 60 U Klenow fragment exo- (Thermo Fisher Scientific), and, finally, purified with Microspin G-50 columns (GE Healthcare); 0.25 mg (western blot) or 0.75 mg (MS) Dynabeads MyOne streptavidin C1 (Thermo Fisher Scientific) was washed twice with WB1000 buffer (1 M NaCl, 20 mM Tris, 0.1% IGEPAL CA-630, and 1 mM EDTA). The purified DNA probes were then immobilized on the streptavidin-coated magnetic beads for 30 min at RT on the rotating wheel. One hundred micrograms (western blot) or 300 μg (MS) of nuclear protein extracts alongside 20 μg (western blot) or 40 μg (MS) of sheared salmon sperm DNA (Thermo Fisher Scientific), diluted in protein-binding buffer (PBB; 50 mM Tris, 150 mM NaCl, 0.25% IGEPAL CA-630, 5 mM MgCl_2_, 1 mM DTT, and 1× cOmplete protease inhibitor; Merck), was added and incubated on the rotating wheel for 2 h at 4°C. The beads were washed thrice with PBB, and bound protein were eluted in 2× Laemmli buffer and boiled for 5 min at 95°C. Protein samples were separated on a 4%–12% NuPAGE 4%–12% Bis-Tris protein gels (Thermo Fisher Scientific) or 12% Bis-Tris protein gel (Thermo Fisher Scientific) for 1 h (western blot) or 30 min (MS) at 170 V in 1× NuPAGE MOPS SDS running buffer (Thermo Fisher Scientific). The colloidal blue staining kit (Thermo Fisher Scientific) was used to stain protein samples destined for downstream mass spectrometry analysis.

### Western blot

Cells were lysed in RIPA buffer supplemented with cOmplete protease inhibitor (Merck) by incubation on ice for 15 min. After centrifugation at 14,000*g*, the supernatant was transferred to a fresh tube and quantified with a Pierce BCA protein assay kit (Thermo Fisher Scientific). Thirty to 50 μg of protein extract was diluted in LDS sample buffer supplemented with 0.1 M DTT and boiled for 10 min at 70°C. Protein samples were separated on a 4%–12% NuPAGE 4%–12% Bis-Tris protein gels (Thermo Fisher Scientific) for 1 h at 170 V in 1× NuPAGE MOPS SDS running buffer (Thermo Fisher Scientific). Separated proteins were transferred from the gel to a methanol-activated PVDF membrane for between 1 and 1.5 h at 70–220 mAmp. The membranes were blocked for 1 h at RT in PBS containing 5% (w/v) skim milk (Nacalai Tesque) and 0.1% Tween 20 (Nacalai Tesque; PBS-T) and incubated with primary antibodies overnight at 4°C or for 1 h at RT. Antibodies used are listed in Supplemental Table S9. Membranes were washed thrice in PBS-T and incubated with a secondary antibody for 1 h at RT. Chemiluminescence detection was performed using Pierce ECL western blotting substrate (Thermo Fisher Scientific) or Amersham ECL prime western blotting detection reagent (GE Healthcare).

### Mass spectrometry sample preparation, data acquisition, and analysis

Following gel electrophoresis for 30 min, each lane was separated into four fractions. Gel pieces were then cut into ∼1 × 1-mm pieces and destained twice with destaining buffer (50 mM NH_4_HCO_3_ and 50% ethanol), followed by dehydration with acetonitrile (ACN), and were dried further in a speed vac. Reduction buffer (50 mM NH_4_HCO_3_, 10 mM DTT) was then added to rehydrate and reduce the samples for 1 h at 56°C. Reduction buffer was then replaced with alkylation buffer (50 mM NH_4_HCO_3_ and 55 mM ICH_2_CONH_2_; Sigma-Aldrich), and samples were alkylated in the dark for 45 min at RT. The gel pieces were then washed with ABC buffer (50 mM NH_4_HCO_3_), ACN, ABC buffer, and ACN twice again, before drying further with a speed vac until the pieces were “dice-like bouncy.” The protein samples were then incubated in digestion buffer (50 mM NH_4_HCO_3_ and 10 μg/mL sequencing grade modified trypsin; Promega) overnight at 37°C. Proteins were extracted, and the supernatants were combined by incubating gel pieces in extraction buffer (35 mM ABC buffer, 30% ACN, 3% trifluoroacetic acid), ACN, extraction buffer, and ACN twice again. The combined supernatants were concentrated in a speed vac (Eppendorf) for ∼2 h before being transferred to pre-equilibrated stage tips. Stage tips were prepared by installing two C18 layers (Waters) inside a 200-μL pipette tip. The tips were washed with methanol and 80% ACN supplemented with 0.5% formic acid and then equilibrated by washing with 0.5% formic acid twice. After loading the combined supernatants, the tips were centrifuged followed by a wash with 0.5% formic acid. Tryptic peptides were eluted from stage tips with 80% ACN supplemented with 0.5% formic acid into a plate. Peptides were analyzed by nanoflow liquid chromatography on an EASY-nLC 1200 system coupled to a Q exactive HF mass spectrometer (Thermo Fisher Scientific). Peptides were separated on a C18-reversed phase column (25 cm long, 75-μm inner diameter) packed in-house with ReproSil-Pur C18-AQ 1.9 μm resin (Dr. Maisch). The column was mounted on an easy flex nano source and temperature-controlled by a column oven (Sonation) at 40°C. A 105-min gradient from 2% to 40% ACN in 0.5% formic acid at a flow of 225 nL/min was used. Spray voltage was set to 2.4 kV. The Q exactive HF was operated with a TOP20 MS/MS spectra acquisition method per MS full scan. MS scans were conducted with 60,000 resolution at a maximum injection time of 20 msec and MS/MS scans with 15,000 resolution at a maximum injection time of 50 msec. The raw files were processed with MaxQuant (Cox and Mann 2008) version 1.5.2.8 with preset standard settings for SILAC-labeled samples, and the requantify option was activated. Carbamidomethylation was set as fixed modification, and methionine oxidation and protein N-acetylation were considered as variable modifications. Search results were filtered with a false-discovery rate of 0.01. Known contaminants, protein groups only identified by site, and reverse hits of the MaxQuant results were removed, and only proteins were kept that were quantified by SILAC ratios in both “forward” and “reverse” samples. The final SILAC plots were created with the aid of an in-house Python script (Supplemental Code; https://github.com/Kappei-Lab/SILAC-Data-Plotting). Heavy SILAC-labeled input material was quality controlled to ensure a SILAC incorporation rate of >98%.

### Telomerase repeat amplification protocol

Cells were lysed with two times the pellet volume of TRAP lysis buffer (1% IGEPAL CA-630, 50 mM Tris, 150 mM NaCl, 1× cOmplete protease inhibitor; Merck) for 30 min on ice. The cells were then pelleted, and the supernatant was transferred to fresh tubes and quantified using the Pierce BCA protein assay kit according to the manufacturer's instructions. Each TRAP-qPCR was set up in a 20 μL reaction, consisting of 10 μL of 2× QuantiNOVA SYBR Green PCR master mix (Qiagen), 7.2 μL nuclease-free water, 0.4 μL of 10 μM (μmol/L) TS primer, and ACX primer each and 2 μL of 250 ng/μL or 25 ng/μL protein sample (500 ng or 50 ng total nuclear extract, respectively). All reactions were performed in triplicates. The PCR protocol was as follows: 30 min at 25°C for reverse transcription by telomerase and then heat inactivation and hot-start of Taq polymerase for 2 min at 95°C, followed by 32 cycles of 5 sec at 95°C (denaturation) and 90 sec at 60°C (annealing and elongation), and hold at 10°C. Cell lysates from telomerase-negative cells (U2OS and Saos2) and heat-inactivated lysates (10 min at 85°C) were used as negative controls, whereas HT-1080 ST lysates were used as positive controls. qPCR analysis was performed using Thermo Fisher Scientific software.

For TRAP-ddPCR, the telomerase extension was performed first, with each reaction being set up with 5 μL of 10× TRAP extension buffer (630 mM KCl, 200 mM Tris, 15 mM MgCl_2_, 0.5% v/v Tween 20, and 10 mM EGTA), 1 μL of 10 μM TS primer, 1 μL of 10 mM dNTP mix, and 500 ng of nuclear protein sample topped up to 40 μL with nuclease-free water. The samples were incubated 30 min at 25°C and 5 min at 95°C and cooled to 4°C. To set up the ddPCR, each reaction consists of 11 μL 2× QX200 ddPCR EvaGreen Supermix; 0.11 μL of 10 μM TS primer and ACX primer each were added to 2.2 μL of telomerase-extended products topped up to 22 μL with nuclease-free water. Oil droplets were generated using the automated droplet generator (Bio-Rad), followed by plate sealing with a pierceable foil heat seal. A PCR was conducted with the following conditions: 5 min at 95°C; followed by 40 cycles of 30 sec at 95°C, 30 sec at 54°C, and 30 sec at 72°C; and, finally, hold at 4°C with a ramp-rate of 2.5°C/sec between each step. Finally, the plate was transferred to a QX200 droplet reader (Bio-Rad), and positive droplets were analyzed using the QuantaSoft software (Bio-Rad).

### Pyrosequencing

For pyrosequencing, 500 ng of gDNA was subjected to bisulfite conversion using the EpiTect bisulfite kit (Qiagen). PCR reactions were performed on 12.5 ng of bisulfite-treated DNA in a final volume of 25 µL using the Pyromark PCR kit (Qiagen) with one of the primers being biotinylated for later capture. The primers were designed using the PyroMark assay design software 2.0 (Qiagen) (Supplemental Table S10). The initial denaturation/activation step was performed for 15 min at 95°C, followed by 50 cycles of 30 sec at 94°C, 30 sec at 54°C, 45 sec at 72°C, and a final extension step for 10 min at 72°C. The quality and the size of the PCR products were evaluated by running 5 µL of each PCR product on 1.5% (w/v) agarose gel in a 0.5× TBE buffer. The biotinylated PCR products were immobilized on streptavidin-coated Sepharose beads (GE Healthcare). DNA strands were separated using the PyroMark Q24 vacuum workstation; the biotinylated single strands were annealed with 0.375 µM sequencing primer (Supplemental Table S10) and used as a template for pyrosequencing. Pyrosequencing was performed using PyroMark Q24 advanced (Qiagen) according to the manufacturer's instructions, and data about methylation at each CpG were extracted and analyzed using the PyroMark Q24 advanced 3.0.0 software (Qiagen).

## Data access

The mass spectrometry proteomics data from this study have been submitted to the ProteomeXchange Consortium via the PRIDE partner repository (Perez-Riverol et al. 2022) with the data set identifier PXD037776.

## Supplementary Material

Supplement 1

Supplement 2

Supplement 3

Supplement 4

Supplement 5

## References

[GR277724CHUC1] Bell RJA, Rube HT, Kreig A, Mancini A, Fouse SD, Nagarajan RP, Choi S, Hong C, He D, Pekmezci M, 2015. The transcription factor GABP selectively binds and activates the mutant TERT promoter in cancer. Science 348: 1036–1039. 10.1126/science.aab0015 25977370 PMC4456397

[GR277724CHUC2] Bell RJA, Rube HT, Xavier-Magalhães A, Costa BM, Mancini A, Song JS, Costello JF. 2016. Understanding TERT promoter mutations: a common path to immortality. Mol Cancer Res 14: 315–323. 10.1158/1541-7786.MCR-16-0003 26941407 PMC4852159

[GR277724CHUC3] Borah S, Xi L, Zaug AJ, Powell NM, Dancik GM, Cohen SB, Costello JC, Theodorescu D, Cech TR. 2015. *TERT* promoter mutations and telomerase reactivation in urothelial cancer. Science 347: 1006–1010. 10.1126/science.1260200 25722414 PMC4640672

[GR277724CHUC4] Butter F, Kappei D, Buchholz F, Vermeulen M, Mann M. 2010. A domesticated transposon mediates the effects of a single-nucleotide polymorphism responsible for enhanced muscle growth. EMBO Rep 11: 305–311. 10.1038/embor.2010.6 20134481 PMC2854606

[GR277724CHUC5] Butter F, Davison L, Viturawong T, Scheibe M, Vermeulen M, Todd JA, Mann M. 2012. Proteome-wide analysis of disease-associated SNPs that show allele-specific transcription factor binding. PLoS Genet 8: e1002982. 10.1371/journal.pgen.1002982 23028375 PMC3459973

[GR277724CHUC6] Casillas MA, Brotherton SL, Andrews LG, Ruppert JM, Tollefsbol TO. 2003. Induction of endogenous telomerase (hTERT) by c-Myc in WI-38 fibroblasts transformed with specific genetic elements. Gene 316: 57–65. 10.1016/S0378-1119(03)00739-X 14563552

[GR277724CHUC7] Chiba K, Lorbeer FK, Shain AH, McSwiggen DT, Schruf E, Oh A, Ryu J, Darzacq X, Bastian BC, Hockemeyer D. 2017. Mutations in the promoter of the telomerase gene T*ERT* contribute to tumorigenesis by a two-step mechanism. Science 357: 1416–1420. 10.1126/science.aao0535 28818973 PMC5942222

[GR277724CHUC8] Choi J-H, Min NY, Park J, Kim JH, Park SH, Ko YJ, Kang Y, Moon YJ, Rhee S, Ham SW, 2010. TSA-induced DNMT1 down-regulation represses hTERT expression via recruiting CTCF into demethylated core promoter region of hTERT in HCT116. Biochem Bioph Res Commun 391: 449–454. 10.1016/j.bbrc.2009.11.078 19914212

[GR277724CHUC9] Cong Y-S, Bacchetti S. 2000. Histone deacetylation is involved in the transcriptional repression of hTERT in normal human cells. J Biol Chem 275: 35665–35668. 10.1074/jbc.C000637200 10986277

[GR277724CHUC10] Cong Y-S, Wen J, Bacchetti S. 1999. The human telomerase catalytic subunit hTERT: organization of the gene and characterization of the promoter. Hum Mol Genet 8: 137–142. 10.1093/hmg/8.1.137 9887342

[GR277724CHUC11] Cox J, Mann M. 2008. MaxQuant enables high peptide identification rates, individualized p.p.b.-range mass accuracies and proteome-wide protein quantification. Nat Biotechnol 26: 1367–1372. 10.1038/nbt.1511 19029910

[GR277724CHUC12] Cristofari G, Lingner J. 2006. Telomere length homeostasis requires that telomerase levels are limiting. EMBO J 25: 565–574. 10.1038/sj.emboj.7600952 16424902 PMC1383536

[GR277724CHUC13] Dessain SK, Yu H, Reddel RR, Beijersbergen RL, Weinberg RA. 2000. Methylation of the human telomerase gene CpG island. Cancer Res 60: 537–541. 10676632

[GR277724CHUC14] Esopi D, Graham MK, Brosnan-Cashman JA, Meyers J, Vaghasia A, Gupta A, Kumar B, Haffner MC, Heaphy CM, Marzo AMD, 2020. Pervasive promoter hypermethylation of silenced *TERT* alleles in human cancers. Cell Oncol 43: 847–861. 10.1007/s13402-020-00531-7 PMC7581602 32468444

[GR277724CHUC15] Fang J, Jia J, Makowski M, Xu M, Wang Z, Zhang T, Hoskins JW, Choi J, Han Y, Zhang M, 2017. Functional characterization of a multi-cancer risk locus on chr5p15.33 reveals regulation of *TERT* by ZNF148. Nat Commun 8: 15034. 10.1038/ncomms15034 28447668 PMC5414179

[GR277724CHUC16] Fernandes ER, Rooney RJ. 1997. The adenovirus E1A-regulated transcription factor E4F is generated from the human homolog of nuclear factor ϕAP3. Mol Cell Biol 17: 1890–1903. 10.1128/MCB.17.4.1890 9121437 PMC232036

[GR277724CHUC17] Ge Z, Li W, Wang N, Liu C, Zhu Q, Björkholm M, Gruber A, Xu D. 2010. Chromatin remodeling: recruitment of histone demethylase RBP2 by Madl for transcriptional repression of a Myc target gene, telomerase reverse transcriptase. FASEB J 24: 579–586. 10.1096/fj.09-140087 19762557

[GR277724CHUC18] Hanahan D, Weinberg RA. 2011. Hallmarks of cancer: the next generation. Cell 144: 646–674. 10.1016/j.cell.2011.02.013 21376230

[GR277724CHUC19] Hollenhorst PC, Shah AA, Hopkins C, Graves BJ. 2007. Genome-wide analyses reveal properties of redundant and specific promoter occupancy within the *ETS* gene family. Gene Dev 21: 1882–1894. 10.1101/gad.1561707 17652178 PMC1935027

[GR277724CHUC20] Horikawa I, Cable PL, Afshari C, Barrett JC. 1999. Cloning and characterization of the promoter region of human telomerase reverse transcriptase gene. Cancer Res 59: 826–830. 10029071

[GR277724CHUC21] Horn S, Figl A, Rachakonda PS, Fischer C, Sucker A, Gast A, Kadel S, Moll I, Nagore E, Hemminki K, 2013. *TERT* promoter mutations in familial and sporadic melanoma. Science 339: 959–961. 10.1126/science.1230062 23348503

[GR277724CHUC22] Hou M, Wang X, Popov N, Zhang A, Zhao X, Zhou R, Zetterberg A, Björkholm M, Henriksson M, Gruber A, 2002. The histone deacetylase inhibitor trichostatin a derepresses the telomerase reverse transcriptase (hTERT) gene in human cells. Exp Cell Res 274: 25–34. 10.1006/excr.2001.5462 11855854

[GR277724CHUC23] Huang FW, Hodis E, Xu MJ, Kryukov GV, Chin L, Garraway LA. 2013. Highly recurrent *TERT* promoter mutations in human melanoma. Science 339: 957–959. 10.1126/science.1229259 23348506 PMC4423787

[GR277724CHUC24] Hunter SA, Iwei Y, Ivanka K, Aravindhan S, Eric T, Alexander G, Reinhard D, Jeffrey N, Laura P, Beth R, 2015. The genetic evolution of melanoma from precursor lesions. New Engl J Med 373: 1926–1936. 10.1056/NEJMoa1502583 26559571

[GR277724CHUC25] Hurst CD, Platt FM, Knowles MA. 2014. Comprehensive mutation analysis of the TERT promoter in bladder cancer and detection of mutations in voided urine. Eur Urol 65: 367–369. 10.1016/j.eururo.2013.08.057 24035680

[GR277724CHUC26] Kappei D, Butter F, Benda C, Scheibe M, Draškovič I, Stevense M, Novo CL, Basquin C, Araki M, Araki K, 2013. HOT1 is a mammalian direct telomere repeat-binding protein contributing to telomerase recruitment. EMBO J 32: 1681–1701. 10.1038/emboj.2013.105 23685356 PMC3680732

[GR277724CHUC27] Kappei D, Scheibe M, Paszkowski-Rogacz M, Bluhm A, Gossmann TI, Dietz S, Dejung M, Herlyn H, Buchholz F, Mann M, 2017. Phylointeractomics reconstructs functional evolution of protein binding. Nat Commun 8: 14334. 10.1038/ncomms14334 28176777 PMC5309834

[GR277724CHUC28] Killela PJ, Reitman ZJ, Jiao Y, Bettegowda C, Agrawal N, Diaz LA, Friedman AH, Friedman H, Gallia GL, Giovanella BC, 2013. *TERT* promoter mutations occur frequently in gliomas and a subset of tumors derived from cells with low rates of self-renewal. Proc Natl Acad Sci 110: 6021–6026. 10.1073/pnas.1303607110 23530248 PMC3625331

[GR277724CHUC29] Kinde I, Munari E, Faraj SF, Hruban RH, Schoenberg M, Bivalacqua T, Allaf M, Springer S, Wang Y, Diaz LA, 2013. *TERT* promoter mutations occur early in urothelial neoplasia and are biomarkers of early disease and disease recurrence in urine. Cancer Res 73: 7162–7167. 10.1158/0008-5472.CAN-13-2498 24121487 PMC3966102

[GR277724CHUC30] Kyo S, Takakura M, Fujiwara T, Inoue M. 2008. Understanding and exploiting *hTERT* promoter regulation for diagnosis and treatment of human cancers. Cancer Sci 99: 1528–1538. 10.1111/j.1349-7006.2008.00878.x 18754863 PMC11158053

[GR277724CHUC31] Lambert SA, Jolma A, Campitelli LF, Das PK, Yin Y, Albu M, Chen X, Taipale J, Hughes TR, Weirauch MT. 2018. The human transcription factors. Cell 172: 650–665. 10.1016/j.cell.2018.01.029 29425488 PMC12908702

[GR277724CHUC32] Le Cam L, Linares LK, Paul C, Julien E, Lacroix M, Hatchi E, Triboulet R, Bossis G, Shmueli A, Rodriguez MS, 2006. E4F1 is an atypical ubiquitin ligase that modulates p53 effector functions independently of degradation. Cell 127: 775–788. 10.1016/j.cell.2006.09.031 17110336

[GR277724CHUC33] Lee KA, Green MR. 1987. A cellular transcription factor E4F1 interacts with an E1a-inducible enhancer and mediates constitutive enhancer function in vitro. EMBO J 6: 1345–1353. 10.1002/j.1460-2075.1987.tb02374.x 2956091 PMC553939

[GR277724CHUC34] Lee JH, Lee JE, Kahng JY, Kim SH, Park JS, Yoon SJ, Um J-Y, Kim WK, Lee J-K, Park J, 2017. Human glioblastoma arises from subventricular zone cells with low-level driver mutations. Nature 560: 243–247. 10.1038/s41586-018-0389-3 30069053

[GR277724CHUC35] Li Y, Zhou Q-L, Sun W, Chandrasekharan P, Cheng HS, Ying Z, Lakshmanan M, Raju A, Tenen DG, Cheng S-Y, 2015. Non-canonical NF-κB signalling and ETS1/2 cooperatively drive C250T mutant TERT promoter activation. Nat Cell Biol 17: 1327–1338. 10.1038/ncb3240 26389665 PMC4772727

[GR277724CHUC36] Liu C, Fang X, Ge Z, Jalink M, Kyo S, Björkholm M, Gruber A, Sjöberg J, Xu D. 2007. The *Telomerase Reverse Transcriptase* (*hTERT*) gene is a direct target of the histone methyltransferase SMYD3. Cancer Res 67: 2626–2631. 10.1158/0008-5472.CAN-06-4126 17363582

[GR277724CHUC37] Liu NQ, Huurne MT, Nguyen LN, Peng T, Wang S-Y, Studd JB, Joshi O, Ongen H, Bramsen JB, Yan J, 2017. The non-coding variant rs1800734 enhances DCLK3 expression through long-range interaction and promotes colorectal cancer progression. Nat Commun 8: 14418. 10.1038/ncomms14418 28195176 PMC5316867

[GR277724CHUC38] Lorbeer FK, Hockemeyer D. 2020. TERT promoter mutations and telomeres during tumorigenesis. Curr Opin Genet Dev 60: 56–62. 10.1016/j.gde.2020.02.001 32163830

[GR277724CHUC39] Ludlow AT, Robin JD, Sayed M, Litterst CM, Shelton DN, Shay JW, Wright WE. 2014. Quantitative telomerase enzyme activity determination using droplet digital PCR with single cell resolution. Nucleic Acids Res 42: e104–e104. 10.1093/nar/gku439 24861623 PMC4117742

[GR277724CHUC40] Ludlow AT, Shelton D, Wright WE, Shay JW. 2018. Digital PCR, methods and protocols. Methods Mol Biol 1768: 513–529. 10.1007/978-1-4939-7778-9_29 29717462 PMC6046637

[GR277724CHUC41] Makowski MM, Willems E, Fang J, Choi J, Zhang T, Jansen PWTC, Brown KM, Vermeulen M. 2016. An interaction proteomics survey of transcription factor binding at recurrent TERT promoter mutations. Proteomics 16: 417–426. 10.1002/pmic.201500327 26553150

[GR277724CHUC42] Mancini A, Xavier-Magalhães A, Woods WS, Nguyen K-T, Amen AM, Hayes JL, Fellmann C, Gapinske M, McKinney AM, Hong C, 2018. Disruption of the β1L isoform of GABP reverses glioblastoma replicative immortality in a TERT promoter mutation-dependent manner. Cancer Cell 34: 513–528.e8. 10.1016/j.ccell.2018.08.003 30205050 PMC6135086

[GR277724CHUC43] Mondal S, Ramanathan M, Miao W, Meyers RM, Rao D, Lopez-Pajares V, Siprashvili Z, Reynolds DL, Porter DF, Ferguson I, 2022. PROBER identifies proteins associated with programmable sequence-specific DNA in living cells. Nat Methods 19: 959–968. 10.1038/s41592-022-01552-w 35927480 PMC10202087

[GR277724CHUC44] Nault JC, Mallet M, Pilati C, Calderaro J, Bioulac-Sage P, Laurent C, Laurent A, Cherqui D, Balabaud C, Zucman-Rossi J, 2013. High frequency of telomerase reverse-transcriptase promoter somatic mutations in hepatocellular carcinoma and preneoplastic lesions. Nat Commun 4: 2218. 10.1038/ncomms3218 23887712 PMC3731665

[GR277724CHUC45] Oikawa T, Yamada T. 2003. Molecular biology of the Ets family of transcription factors. Gene 303: 11–34. 10.1016/S0378-1119(02)01156-3 12559563

[GR277724CHUC46] Olivier M, Hollstein M, Hainaut P. 2010. TP53 mutations in human cancers: origins, consequences, and clinical use. CSH Perspect Biol 2: a001008. 10.1101/cshperspect.a001008 PMC2827900 20182602

[GR277724CHUC47] Perez-Riverol Y, Bai J, Bandla C, García-Seisdedos D, Hewapathirana S, Kamatchinathan S, Kundu DJ, Prakash A, Frericks-Zipper A, Eisenacher M, 2022. The PRIDE database resources in 2022: a hub for mass spectrometry-based proteomics evidences. Nucleic Acids Res 50: D543–D552. 10.1093/nar/gkab1038 34723319 PMC8728295

[GR277724CHUC48] Rachakonda S, Hoheisel JD, Kumar R. 2021. Occurrence, functionality and abundance of the *TERT* promoter mutations. Int J Cancer 149: 1852–1862. 10.1002/ijc.33750 34313327

[GR277724CHUC49] Rajagopalan D, Pandey AK, Xiuzhen MC, Lee KK, Hora S, Zhang Y, Chua BH, Kwok HS, Bhatia SS, Deng LW, 2017. TIP60 represses telomerase expression by inhibiting Sp1 binding to the TERT promoter. PLoS Pathog 13: e1006681. 10.1371/journal.ppat.1006681 29045464 PMC5662243

[GR277724CHUC50] Renaud S, Loukinov D, Abdullaev Z, Guilleret I, Bosman FT, Lobanenkov V, Benhattar J. 2007. Dual role of DNA methylation inside and outside of CTCF-binding regions in the transcriptional regulation of the telomerase hTERT gene. Nucleic Acids Res 35: 1245–1256. 10.1093/nar/gkl1125 17267411 PMC1851636

[GR277724CHUC51] Rodier G, Kirsh O, Baraibar M, Houlès T, Lacroix M, Delpech H, Hatchi E, Arnould S, Severac D, Dubois E, 2015. The transcription factor E4F1 coordinates CHK1-dependent checkpoint and mitochondrial functions. Cell Rep 11: 220–233. 10.1016/j.celrep.2015.03.024 25843721

[GR277724CHUC52] Rooney RJ, Rothammer K, Fernandes ER. 1998. Mutational analysis of p50E4F suggests that DNA binding activity is mediated through an alternative structure in a zinc finger domain that is regulated by phosphorylation. Nucleic Acids Res 26: 1681–1688. 10.1093/nar/26.7.1681 9512539 PMC147461

[GR277724CHUC53] Shay JW, Bacchetti S. 1997. A survey of telomerase activity in human cancer. Eur J Cancer 33: 787–791. 10.1016/S0959-8049(97)00062-2 9282118

[GR277724CHUC54] Stern JL, Theodorescu D, Vogelstein B, Papadopoulos N, Cech TR. 2015. Mutation of the *TERT* promoter, switch to active chromatin, and monoallelic *TERT* expression in multiple cancers. Genes Dev 29: 2219–2224. 10.1101/gad.269498.115 26515115 PMC4647555

[GR277724CHUC55] Stern JL, Paucek RD, Huang FW, Ghandi M, Nwumeh R, Costello JC, Cech TR. 2017. Allele-specific DNA methylation and its interplay with repressive histone marks at promoter-mutant TERT genes. Cell Rep 21: 3700–3707. 10.1016/j.celrep.2017.12.001 29281820 PMC5747321

[GR277724CHUC56] Takakura M, Kyo S, Kanaya T, Hirano H, Takeda J, Yutsudo M, Inoue M. 1999. Cloning of human telomerase catalytic subunit (hTERT) gene promoter and identification of proximal core promoter sequences essential for transcriptional activation in immortalized and cancer cells. Cancer Res 59: 551–557. 9973199

[GR277724CHUC57] Takakura M, Kyo S, Sowa Y, Wang Z, Yatabe N, Maida Y, Tanaka M, Inoue M. 2001. Telomerase activation by histone deacetylase inhibitor in normal cells. Nucleic Acids Res 29: 3006–3011. 10.1093/nar/29.14.3006 11452025 PMC55810

[GR277724CHUC58] Vallarelli AF, Rachakonda PS, André J, Heidenreich B, Riffaud L, Bensussan A, Kumar R, Dumaz N. 2016. TERT promoter mutations in melanoma render TERT expression dependent on MAPK pathway activation. Oncotarget 7: 53127–53136. 10.18632/oncotarget.10634 27449293 PMC5288173

[GR277724CHUC59] Vinagre J, Almeida A, Pópulo H, Batista R, Lyra J, Pinto V, Coelho R, Celestino R, Prazeres H, Lima L, 2013. Frequency of TERT promoter mutations in human cancers. Nat Commun 4: 2185. 10.1038/ncomms3185 23887589

[GR277724CHUC60] Wang Z, Zhang Q. 2009. Genome-wide identification and evolutionary analysis of the animal specific ETS transcription factor family. Evol Bioinform 5: EBO.S2948. 10.4137/EBO.S2948 PMC2789578 20011068

[GR277724CHUC61] Wei G, Badis G, Berger MF, Kivioja T, Palin K, Enge M, Bonke M, Jolma A, Varjosalo M, Gehrke AR, 2010. Genome-wide analysis of ETS-family DNA-binding *in vitro* and *in vivo*. EMBO J 29: 2147–2160. 10.1038/emboj.2010.106 20517297 PMC2905244

[GR277724CHUC62] Wu K-J, Grandori C, Amacker M, Simon-Vermot N, Polack A, Lingner J, Dalla-Favera R. 1999. Direct activation of TERT transcription by c-MYC. Nat Genet 21: 220–224. 10.1038/6010 9988278

[GR277724CHUC63] Xuan Lin QX, Sian S, An O, Thieffry D, Jha S, Benoukraf T. 2018. MethMotif: an integrative cell specific database of transcription factor binding motifs coupled with DNA methylation profiles. Nucleic Acids Res 47 (D1)**:** D145–D154. 10.1093/nar/gky1005 PMC6323897 30380113

[GR277724CHUC64] Zhang Y, Zhang A, Shen C, Zhang B, Rao Z, Wang R, Yang S, Ning S, Mao G, Fang D. 2014. E2F1 acts as a negative feedback regulator of c-Myc-induced hTERT transcription during tumorigenesis. Oncol Rep 32: 1273–1280. 10.3892/or.2014.3287 24969314

