# Supplementary material for: E4F1 and ZNF148 are transcriptional activators of the −57A > C and wild-type TERT promoter
Source: Genome Res. 2023 Nov;33(11):1893–905. doi: 10.1101/gr.277724.123 (PMC10760450; doi:10.1101/gr.277724.123)
Supplement: Supplement 2 [file Supplemental_Information.pdf]

Supplemental Information for

E4F1 and ZNF148 are transcriptional activators of the -57A>C and wild-type *TERT* promoter

Boon Haow Chua[1,2], Nurkaiyisah Zaal Anuar[1], Laure Ferry[3], Cecilia Domrane[3], Anna Wittek[1], Vineeth Mukundan[1], Sudhakar Jha[1,2,4,5], Falk Butter[6], Daniel G. Tenen[1,7], Pierre-Antoine Defossez[3], Dennis Kappei[1,2,4,*]

[1] Cancer Science Institute of Singapore, National University of Singapore, 117599 Singapore
[2] Department of Biochemistry, Yong Loo Lin School of Medicine, National University of Singapore, 117596 Singapore
[3] Université Paris Cité, CNRS, Epigenetics and Cell Fate, F-75013 Paris, France
[4] NUS Center for Cancer Research, Yong Loo Lin School of Medicine, National University of Singapore, Singapore
[5] Department of Physiological Sciences, College of Veterinary Medicine, Oklahoma State University, OK 74078, USA
[6] Institute of Molecular Biology (IMB), Ackermannweg 4, 55128 Mainz, Germany
[7] Harvard Stem Cell Institute, Harvard Medical School, Boston, MA 02115, USA
* To whom correspondence should be addressed. Email: dennis.kappei@nus.edu.sg

Content:

Supplemental Figures S1-3

Supplemental Tables S1-10

Supplemental Code

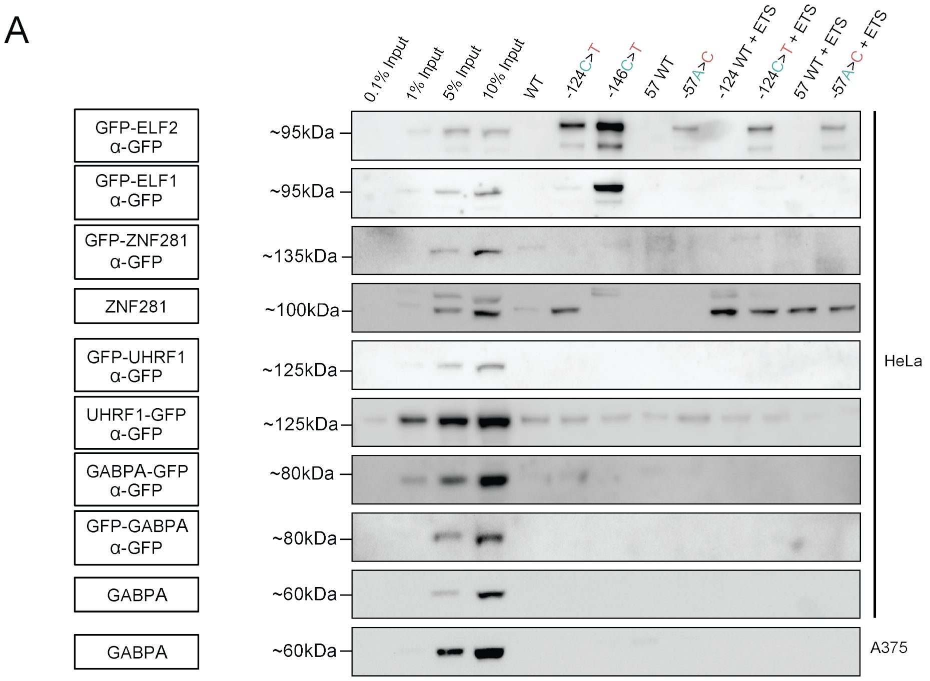

Supplemental Figure S1. (A) Sequence specific pull-down of endogenous and/or recombinant GFP-tagged ELF1, ELF2, ZNF281, UHRF1 and GABPA with HeLa and/or A375 nuclear extracts using the 9 probes shown in Fig. 1A.

A

DNA methylation across the *TERT* promoter

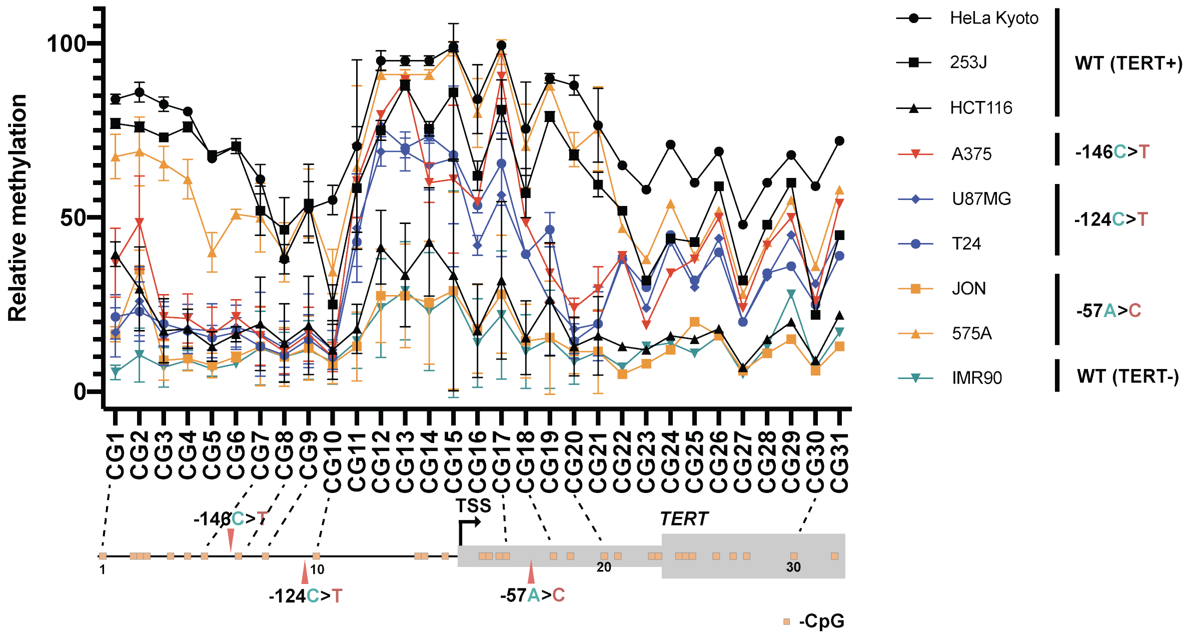

B

DNA methylation across the *TERT* promoter

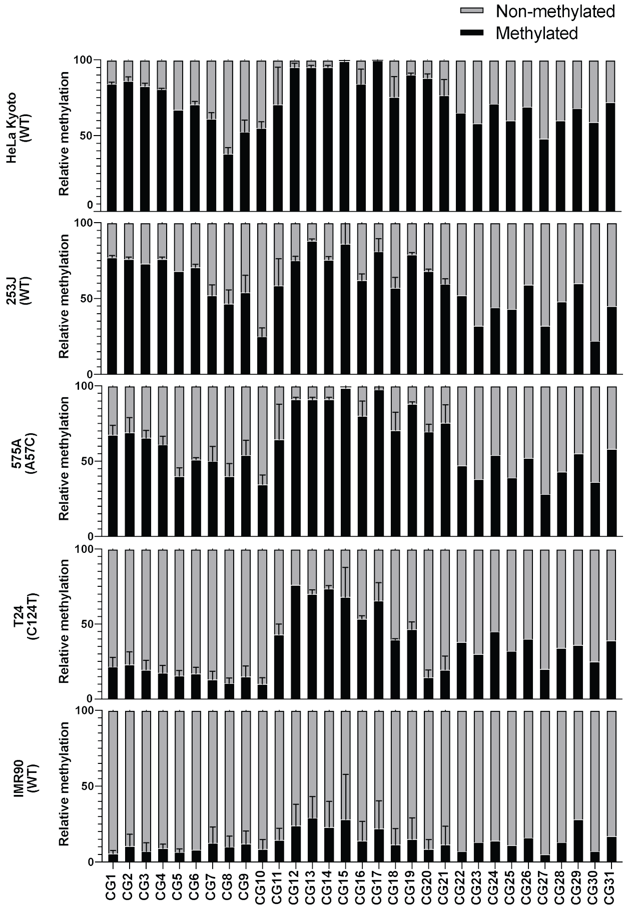

Supplemental Figure S2. (A) Relative DNA methylation frequency of CpGs across the *TERT* promoter in telomerase-positive cell lines with the WT promoter (HeLa Kyoto, 253J, HCT116; black), the -146C>T mutation (A375; red), the -124C>T mutation (U87MG & T24; blue), the -57A>C mutation (575A & JON; orange) or telomerase-negative, non-cancerous cells (IMR-90; green). **(B)** DNA methylation on CpGs across the *TERT* promoter in selected cell lines. Generally, telomerase-positive cell lines exhibit higher methylation levels compared to the primary IMR-90 fibroblasts.

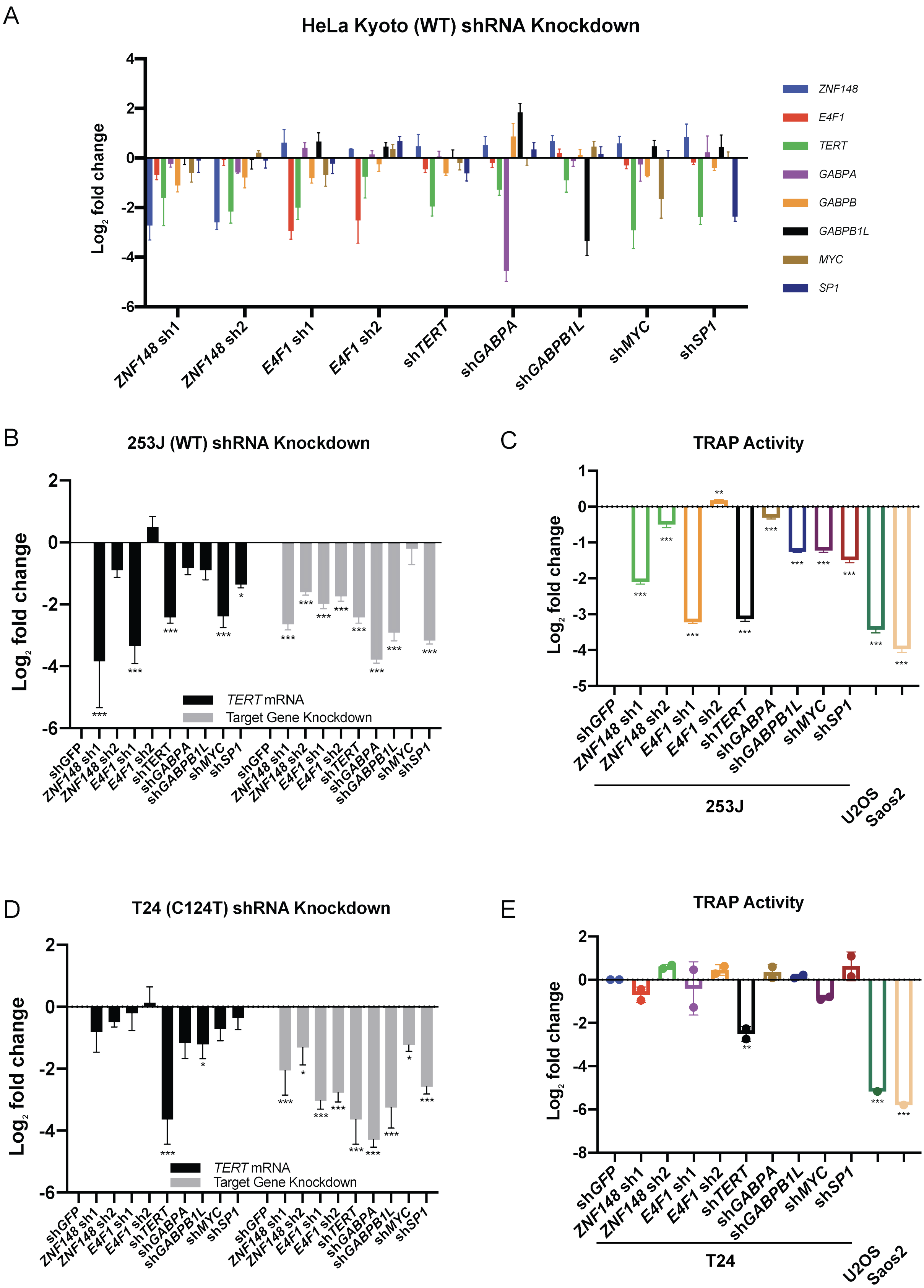
A
HeLa Kyoto (WT) shRNA Knockdown
Log2 fold change
ZNF148
E4F1
TERT
GABPA
GABPB
GABPB1L
MYC
SP1
ZNF148 sh1
ZNF148 sh2
E4F1 sh1
E4F1 sh2
shTERT
shGABPA
shGABPB1L
shMYC
shSP1
B
253J (WT) shRNA Knockdown
TERT mRNA
Target Gene Knockdown
C
TRAP Activity
shGFP
253J
U2OS
Saos2
D
T24 (C124T) shRNA Knockdown
E
TRAP Activity
T24

Supplemental Figure S3. (A) mRNA expression data of *ZNF148*, *E4F1*, *TERT*, *GABPA*, *GABPB1L*, *MYC* and *SP1* following 48+72 h post-shRNA knockdown in HeLa (48h virus transduction, 72 hours puromycin selection), with sh*GFP* as control. Data shown as mean of values from three biological replicates. **(B)** mRNA expression data of *TERT* and target gene following 48+72 h post-shRNA knockdown in 253J, with sh*GFP* as control. **(C)** TRAP assay measuring telomerase activity following 48+72 h post-shRNA knockdown in 253J (48h virus transduction, 72 hours puromycin selection), with sh*GFP* as control. Telomerase-negative U2OS and Saos2 were used as negative controls. **(D)** mRNA expression data of *TERT* and target genes following 48+72 h post-shRNA knockdown in T24 (48h virus transduction, 72 hours puromycin selection), with sh*GFP* as control. Data shown as mean of values from three biological replicates. **(E)** ddPCR-TRAP assay measuring telomerase activity following 48+72 h post-shRNA knockdown in T24, with sh*GFP* as control. Telomerase-negative U2OS and Saos2 were used as negative controls. Data shown as mean of values from two biological replicates. All statistical significance was calculated using a two-sampled t-test, and the degree of significance is indicated as: * for $p < 0.05$; ** for $p < 0.01$; *** for $p < 0.001$.

Supplemental Tables

Supplemental Table S1. List of oligonucleotides used for DNA pulldown.

No	Primers	Sequence (5' → 3')
1a	*TERT* promoter WT forward	CTGGGAGGGCCCGGA**G**GGGGCTGGGCCGGGGACCCGG**G**AGGGGTCGGGACGGGGCG
1b	*TERT* promoter WT reverse	AGCGCCCCGTCCCGACCCCT**C**CCGGGTCCCCGGCCCAGCCCC**C**TCCGGGCCCTCCC
2a	*TERT* promoter -124C>T forward	CTGGGAGGGCCCGGA**A**GGGGCTGGGCCGGGGACCCGG**G**AGGGGTCGGGACGGGGCG
2b	*TERT* promoter -124C>T reverse	AGCGCCCCGTCCCGACCCCT**C**CCGGGTCCCCGGCCCAGCCCC**T**TCCGGGCCCTCCC
3a	*TERT* promoter -146C>T forward	CTGGGAGGGCCCGGA**G**GGGGCTGGGCCGGGGACCCGG**A**AGGGGTCGGGACGGGGCG
3b	*TERT* promoter -146C>T reverse	AGCGCCCCGTCCCGACCCCT**T**CCGGGTCCCCGGCCCAGCCCC**C**TCCGGGCCCTCCC
4a	*TERT* promoter -57WT forward	CTCCTCGCGGCGCGAGTTTC**A**GGCAGCGCTGCGTCCTGCTGCGCACGTGGGA
4b	*TERT* promoter -57WT reverse	AGTCCCACGTGCGCAGCAGGACGCAGCGCTGCC**T**GAAACTCGCGCCGCGAGG
5a	*TERT* promoter -57A>C forward	CTCCTCGCGGCGCGAGTTTC**C**GGCAGCGCTGCGTCCTGCTGCGCACGTGGGA
5b	*TERT* promoter -57A>C reverse	AGTCCCACGTGCGCAGCAGGACGCAGCGCTGCC**G**GAAACTCGCGCCGCGAGG
6a	*TERT* promoter wt(124)+ETS96+ETS91 forward	ACCGGGGCCGCGGAAAGGAAGGGGAGGGGCTGGGAGGGCCCGGA**G**GGGGCTGGGCCGGGG
6b	*TERT* promoter wt(124)+ETS96+ETS91 reverse	GTCCCCGGCCCAGCCCC**C**TCCGGGCCCTCCCAGCCCCTCCCCTTCCTTTCCGCGGCCCCG
7a	*TERT* promoter mt(-124C>T) ETS96+ETS91 forward	ACCGGGGCCGCGGAAAGGAAGGGGAGGGGCTGGGAGGGCCCGGA**A**GGGGCTGGGCCGGGG
7b	*TERT* promoter mt(-124C>T)+ETS96+ETS91 reverse	GTCCCCGGCCCAGCCCC**T**TCCGGGCCCTCCCAGCCCCTCCCCTTCCTTTCCGCGGCCCCG
8a	*TERT* promoter wt(57)+ETS96+ETS91 forward	AGGCAGCGCTGCC**T**GAAACTCGCGCCGCGAGGAGAGGGCGGGGCCGCGGAAAGGAAGGGG
8b	*TERT* promoter wt(57)+ETS96+ETS91 reverse	CTCCCCTTCCTTTCCGCGGCCCCGCCCTCTCCTCGCGGCGCGAGTTTC**A**GGCAGCGCTGC
9a	*TERT* promoter mt(-57A>C)+ETS96+ETS91 forward	AGGCAGCGCTGCC**G**GAAACTCGCGCCGCGAGGAGAGGGCGGGGCCGCGGAAAGGAAGGGG
9b	*TERT* promoter mt(-57A>C)+ETS96+ETS91 reverse	CTCCCCTTCCTTTCCGCGGCCCCGCCCTCTCCTCGCGGCGCGAGTTTC**C**GGCAGCGCTGC
10a	rs36115365 major SNP forward	AGACAGGAGGAAATGGTCTCAGCCTCACC**G**TCCGTGGCCACGGCAGCTTCAGTGAGCCAG
10b	rs36115365 major SNP reverse	CTCTGGCTCACTGAAGCTGCCGTGGCCACGGA**C**GGTGAGGCTGAGACCATTTCCTCCTGT
11a	rs36115365 minor SNP forward	AGACAGGAGGAAATGGTCTCAGCCTCACC**C**TCCGTGGCCACGGCAGCTTCAGTGAGCCAG
11b	rs36115365 minor SNP reverse	CTCTGGCTCACTGAAGCTGCCGTGGCCACGGA**G**GGTGAGGCTGAGACCATTTCCTCCTGT
12a	ZNF148 binding motif forward	GTCCAGCGCACCAACGCAGGCGAGGGACTGGGGGAGGAGGGAAGTGCCCTCCTGCAGCAC
12b	ZNF148 binding motif reverse	ACGTGCTGCAGGAGGGCACTTCCCTCCTCCCCCAGTCCCTCGCCTGCGTTGGTGCGCTGG
13a	rs509813 major SNP forward	CTCACAAAGGCACACTGTTTCCTTGGGCTCCTCCC**C**CCACCCAACCTTAGAGCCCCCAGC
13b	rs509813 major SNP reverse	AGGCTGGGGGCTCTAAGGTTGGGTGG**G**GGGAGGAGCCCAAGGAAACAGTGTGCCTTTGTG
14a	rs509813 minor SNP forward	CTCACAAAGGCACACTGTTTCCTTGGGCTCCTCCC**G**CCACCCAACCTTAGAGCCCCCAGC
14b	rs509813 minor SNP reverse	AGGCTGGGGGCTCTAAGGTTGGGTGG**C**GGGAGGAGCCCAAGGAAACAGTGTGCCTTTGTG

Supplemental Table S2. *TERT* promoter mutation status at different positions of various cell lines.

No	Primers	-146	-124	-57
1	HeLa Kyoto	100% C	100% C	100% A
2	HCT116	100% C	100% C	100% A
3	253J	100% C	100% C	100% A
4	A375	20% C, 80% T	100% C	100% A
5	U87MG	100% C	15% C, 85% T	100% A
6	T24	100% C	100% T	100% A
7	JON	100% C	100% C	40% A, 60% C
8	575A	100% C	100% C	80% A, 20% C

Supplemental Table S3. MS data for SILAC-based in-vitro DNA reconstitution pull-downs using U87MG nuclear extract with -124C>T vs. WT *TERT* promoter probes.

Supplemental Table S4. MS data for SILAC-based in-vitro DNA reconstitution pull-downs using U87MG nuclear extract with -146C>T vs. WT *TERT* promoter probes.

Supplemental Table S5. MS data for SILAC-based in-vitro DNA reconstitution pull-downs using U87MG nuclear extract with -57A>C vs. WT *TERT* promoter probes.

Supplemental Table S6. List of oligonucleotides used for cloning and sequencing of cDNA and gDNA sequences.

Primer	Primer sequence (5' → 3')	Size	Anneal	Extend
EGFP pcDNA4 forward (Colony PCR and sequencing)	CATGGTCCTGCTGGAGTTCGTG	-	~65°C	30 sec/ kb
E4F1 cloning forward	ATGGAGGGCGCGATGGCA	2355 bp	72°C	75 sec
E4F1 cloning reverse	CTAGACGATGACCGTCTGCACCT			
E4F1 cloning forward	ATGGAGGGCGCGATGGCA	2352 bp	72°C	75 sec
E4F1 cloning reverse 2	GACGATGACCGTCTGCACCTCC			
GABPA cloning forward	ATGACTAAAAGAGAAGCAGAGGAG	1365 bp	64°C	46 sec
GABPA cloning reverse	TCAATTATCCTTTTCCGTTTGCAG			
GABPA cloning forward	ATGACTAAAAGAGAAGCAGAGGAG	1362 bp	64°C	45 sec
GABPA cloning reverse 2	ATTATCCTTTTCCGTTTGCAGAGA			
UHRF1 cloning forward	ATGTGGATCCAGGTTCGGACC	2379 bp	70°C	76 sec
UHRF1 cloning reverse 2	CCGGCCATTGCCGTAGCC			
ZNF148 cloning forward	ATGAACATTGACGACAAACTG	2385 bp	62°C	75 sec
ZNF148 cloning reverse	TTAGCCAAAAGTCTGGCCAG			
ZNF281 cloning forward	ATGAAAATCGGCAGTGGGTT	2688 bp	66°C	85 sec
ZNF281 cloning reverse	TTACCTGTAACTCTGGCTGGTG			
ZNF281 cloning forward	ATGAAAATCGGCAGTGGGTT	2685 bp	66°C	85 sec
ZNF281 cloning reverse 2	CCTGTAACTCTGGCTGGTGG			
PCR8 381bp forward (Colony PCR and sequencing)	ACAACGTTCAAATCCGCTCC	-	~61°C	30 sec/ kb
TERT promoter sequencing forward primer	GATTCGACCTCTCTCCGCTG	1151 bp	61°C	40 sec
TERT promoter sequencing reverse primer	CTCCCTGACGCTATGGTTCC			

Supplemental Table S7. List of oligonucleotides used for shRNA generation.

No	Gene shRNA primer	Clone ID	Primer sequence (5' → 3')
1a	sh*E4F1* #1 forward	TRCN0000419634	CCGGAGGACGTGGTTGTCAGCAAAGCTCGAGCTTTGCTGACAACCACGTCCTTTTTTG
1b	sh*E4F1* #1 reverse		AATTCAAAAAAGGACGTGGTTGTCAGCAAAGCTCGAGCTTTGCTGACAACCACGTCCT
2a	sh*E4F1* #2 forward	TRCN0000013826	CCGGTGTTCAGCACAAGATTCAGAACTCGAGTTCTGAATCTTGTGCTGAACATTTTTG
2b	sh*E4F1* #2 reverse		AATTCAAAAATGTTCAGCACAAGATTCAGAACTCGAGTTCTGAATCTTGTGCTGAACA
3a	sh*GABPA* forward	TRCN0000018290	CCGGGCTAGAACTTCTTACTGATAACTCGAGTTATCAGTAAGAAGTTCTAGCTTTTTG
3b	sh*GABPA* reverse		AATTCAAAAAGCTAGAACTTCTTACTGATAACTCGAGTTATCAGTAAGAAGTTCTAGC
4a	sh*GABPB1L* forward	-	CCGGGAGAGAAGCTCTTCAGAAACACTCGAGTGTTTCTGAAGAGCTTCTCTCTTTTTG
4b	sh*GABPB1L* reverse		AATTCAAAAAGAGAGAAGCTCTTCAGAAACACTCGAGTGTTTCTGAAGAGCTTCTCTC
5a	sh*MYC* forward	TRCN0000039639	CCGGCCCAAGGTAGTTATCCTTAAACTCGAGTTTAAGGATAACTACCTTGGGTTTTTG
5b	sh*MYC* reverse		AATTCAAAAACCCAAGGTAGTTATCCTTAAACTCGAGTTTAAGGATAACTACCTTGGG
6a	sh*SP1* forward	TRCN0000360587	CCGGAGCCATCATGCCTTGATAAATCTCGAGATTTATCAAGGCATGATGGCTTTTTTG
6b	sh*SP1* reverse		AATTCAAAAAAGCCATCATGCCTTGATAAATCTCGAGATTTATCAAGGCATGATGGCT
7a	sh*TERT* forward	TRCN0000240466	CCGGGACGGTGTGCACCAACATCTACTCGAGTAGATGTTGGTGCACACCGTCTTTTTG
7b	sh*TERT* reverse		AATTCAAAAAGACGGTGTGCACCAACATCTACTCGAGTAGATGTTGGTGCACACCGTC
8a	sh*ZNF148* #1 forward	TRCN0000230462	CCGGAGTACCACGGCATCCATATTACTCGAGTAATATGGATGCCGTGGTACTTTTTTG
8b	sh*ZNF148* #1 reverse		AATTCAAAAAAGTACCACGGCATCCATATTACTCGAGTAATATGGATGCCGTGGTACT
9a	sh*ZNF148* #2 forward	TRCN0000218261	CCGGCCTGTGCATAGTAGTACTAATCTCGAGATTAGTACTACTATGCACAGGTTTTTG
9b	sh*ZNF148* #2 reverse		AATTCAAAAACCTGTGCATAGTAGTACTAATCTCGAGATTAGTACTACTATGCACAGG
10a	sh*GFP* forward	-	CCGGACAACAGCCACAACGTCTATACTCGAGTATAGACGTTGTGGCTGTTGTTTTTTG
10b	sh*GFP* reverse		AATTCAAAAAACAACAGCCACAACGTCTATACTCGAGTATAGACGTTGTGGCTGTTGT

Supplemental Table S8. List of oligonucleotides used for quantiative PCR.

No	Primers	Sequence (5' → 3')	Product size
1a	*E4F1* forward	GCACAGAGAAAATCCGCTTC	139 bp
1b	*E4F1* reverse	GGTGAAGTCTCTATAGGCTCG	
2a	*GABPA* forward	GAGAAACTCAGTCGTGCATTAAG	149 bp
2b	*GABPA* reverse	CATTCTGTGACCAAACGGTTC	
3a	*GABPB* forward	TCCACTTCATCTAGCAGCACA	107 bp
3b	*GABPB* reverse	GTAATGGTGTTCGGTCCACTT	
4a	*GABPB1L* forward	ATTGAAAACCGGGTGGAATC	134 bp
4b	*GABPB1L* reverse	CTGTAGGCCTCTGCTTCCTG	
5a	*MYC* forward	TTCGGGTAGTGGAAAACCAG	108 bp
5b	*MYC* reverse	AGTAGAAATACGGCTGCACC	
6a	*SP1* forward	CTCCAGACCATTAACCTCAGTG	143 bp
6b	*SP1* reverse	TGTATTCCATCACCACCAGC	
7a	*TBP* forward	TTCGGAGAGTTCTGGGATTG	144 bp
7b	*TBP* reverse	CTCATGATTACCGCAGCAAA	
8a	*TERT* forward	TCACGGAGACCACGTTTCAAA	94 bp
8b	*TERT* reverse	TTCAAGTGCTGTCTGATTCCAAT	
9a	*ZNF148* forward	GCTGCCTTTAGAACGAACTATCA	167 bp
9b	*ZNF148* reverse	CCACATTCATCACAGCGAAAT	

Supplemental Table S9. List of antibodies used for Western blot.

Antibody	Company	Catalogue No.	Origin	Dilution used for WB
GFP	Roche	11814460001	mouse monoclonal	1:4,000
MYC	Santa-Cruz	sc-40	mouse monoclonal	1:250
Tubulin	MPI-CPG		mouse monoclonal	1:10,000
E4F1	Santa-Cruz	sc-514718	mouse monoclonal	1:500
GABPA	Santa-Cruz	sc-28312	mouse monoclonal	1:250
GABPB1/2	Santa-Cruz	sc-271571	mouse monoclonal	1:250
GAPDH	Novus Biologicals	NB300-221	mouse monoclonal	1:1,000
SP1	Santa-Cruz	sc-17824	mouse monoclonal	1:250
ZNF148	Santa-Cruz	sc-137171	mouse monoclonal	1:500

Supplemental Table S10. List of oligonucleotides used for pyrosequencing.

No	Primers	Sequence (5' → 3')	Location
1a	Region 1 forward (biotinylated)	GGTGGTAGGGGTTAGGGTTTTTTA	CpGs 1-10 in the *TERT* promoter
1b	Region 1 reverse	TCCTACCCCTTCACCTTCCAA	
1c	Region 1 sequencing primer	CTTCACCTTCCAACT	
2a	Region 2 forward	GGGGTGGTAGGGGTTAGG	CpGs 11-21 in the *TERT* promoter
2b	Region 2 reverse (biotinylated)	TCCTACCCCTTCACCTTCCAA	
2c	Region 2 sequencing primer	GGGGTTAGGGTTTTT	
3a	Region 3 forward	AGGGTAAGTATATTAGGTATTGGGTTATT	CpGs 22-31 in the *TERT* promoter
3b	Region 3 reverse (biotinylated)	AACCCCTCCCCTTCCTTT	
3c	Region 3 sequencing primer	GTGGTTGAGTAGTAGGGA	

Supplemental Code. Python script for plotting of SILAC DNA pull-down data in Fig. 1C-E (also deposited to GitHub, github.com/Kappei-Lab/SILAC-Data-Plotting)
